# Cluster J Mycobacteriophages: Intron Splicing in Capsid and Tail Genes

**DOI:** 10.1371/journal.pone.0069273

**Published:** 2013-07-09

**Authors:** Welkin H. Pope, Deborah Jacobs-Sera, Aaron A. Best, Gregory W. Broussard, Pamela L. Connerly, Rebekah M. Dedrick, Timothy A. Kremer, Susan Offner, Amenawon H. Ogiefo, Marie C. Pizzorno, Kate Rockenbach, Daniel A. Russell, Emily L. Stowe, Joseph Stukey, Sarah A. Thibault, James F. Conway, Roger W. Hendrix, Graham F. Hatfull

**Affiliations:** 1 Department of Biological Sciences, University of Pittsburgh, Pittsburgh, Pennsylvania, United States of America,; 2 Department of Structural Biology, University of Pittsburgh, Pittsburgh, Pennsylvania, United States of America,; 3 Department of Biology, Hope College, Holland, Michigan, United States of America,; 4 Department of Biology, Bucknell University, Lewisburg, Pennsylvania, United States of America,; 5 Lexington High School, Lexington, Massachusetts, United States of America,; 6 School of Natural Sciences, Indiana University Southeast, New Albany, Indiana, United States of America; University of Rochester, United States of America

## Abstract

Bacteriophages isolated on *Mycobacterium smegmatis* mc^2^155 represent many distinct genomes sharing little or no DNA sequence similarity. The genomes are architecturally mosaic and are replete with genes of unknown function. A new group of genomes sharing substantial nucleotide sequences constitute Cluster J. The six mycobacteriophages forming Cluster J are morphologically members of the *Siphoviridae*, but have unusually long genomes ranging from 106.3 to 117 kbp. Reconstruction of the capsid by cryo-electron microscopy of mycobacteriophage BAKA reveals an icosahedral structure with a triangulation number of 13. All six phages are temperate and homoimmune, and prophage establishment involves integration into a tRNA-Leu gene not previously identified as a mycobacterial *attB* site for phage integration. The Cluster J genomes provide two examples of intron splicing within the virion structural genes, one in a major capsid subunit gene, and one in a tail gene. These genomes also contain numerous free-standing HNH homing endonuclease, and comparative analysis reveals how these could contribute to genome mosaicism. The unusual Cluster J genomes provide new insights into phage genome architecture, gene function, capsid structure, gene mobility, intron splicing, and evolution.

## Introduction

Mycobacteriophages are viruses that infect mycobacterial hosts, and the genome sequences of more than 220 phages isolated on *Mycobacterium smegmatis* mc^2^155 have been described [1,2]. These phages correspond to just two morphotypes –*Siphoviridae* and the *Myoviridae* – but many different genome types are represented [3]. To accommodate heterogeneity within their diverse genetic landscape, the mycobacteriophages have been sorted into Clusters according to their overall nucleotide sequence similarity, and there are at least 25 genome types (Clusters A-O, and eight singletons each of which has no close relatives) that share little or no DNA sequence similarity [1,4]. The relationships within clusters are also complex, with genomes in some clusters having few differences, while other clusters can be readily divided into subclusters [5].

The characteristic architectural feature of phage genomes is their pervasive mosaicism, such that individual genomes can be considered as unique assemblages of individual modules, each of which can be recombined within the microbial metagenome [6,7]. As such, genetic modules – which in the mycobacteriophages often correspond to single genes – are found in distinctly different genome contexts, flanked by unrelated genes. The evidence for common ancestry of modules is typically constrained to recognition at the amino acid sequence level, reflecting distant ancestry within a likely very old but dynamic phage population [8]. The mechanisms that generate phage genome mosaicism remain unclear but illegitimate recombination, perhaps mediated by phage recombinases [9], is predicted to play a significant role, with homologous recombination within shared sequences assisting in reassortment of module boundaries [6,7].

Within the mycobacteriophages, a number of different types of mobile elements have been described, all of which could contribute to genome mosaicism. For example, a new class of small transposon-like mycobacteriophage mobile elements (MPME) have been identified through comparative genomics and are present in various genome types [10]. Genes with sequence similarity to IS110-like transposons have also been identified in phages Omega and Bethlehem, although closely related genomes lacking the insertions have yet to be identified [3]. A variety of different inteins have been observed in terminase proteins and in non-structural genes, and there are numerous examples of predicted HNH homing endonucleases [2].

Self-splicing introns – mostly of the group I variety – have been described in many different phages, but predominantly in those that infect either Gram-negative or low GC% Gram-positive bacterial hosts. These include group I introns in the *td*, *nrdD* and *nrdB* genes of T4 related phages, in the DNA Polymerase gene of phage SPOI and its relatives, at least five introns in Staphylococcal phages including in ribonuclease reductase, helicase, and lysis genes [11-14], in the capsid subunit gene of lactococcal phage Tuc2009 [15], and in the *psbA* gene of *Synechococcus* phage S-PM2 [16]. Some, but not all of the introns contain genes encoding homing endonucleases and the intron can be mobilized to an intronless target site by homing endonucleolytic cleavage and repair from the intron-containing allele [17]. However, introns have not been described in mycobacteriophages or any other phages of high GC% Gram-positive bacteria to our knowledge.

Here we describe a comparative genomic analysis of the six mycobacteriophages that constitute Cluster J. These phages are temperate, form a homoimmune group, have unusually long genomes for phages with siphoviral morphotypes, and are packaged into icosahedral capsids with a triangulation number of 13. Among their numerous features of interest, are two distinct types of group I introns; they are found in only three of the six Cluster J genomes – one in a capsid gene and the other two (which are identical) in tail genes – and all efficiently spliced out during late gene expression. The capsid intron contains a putative homing endonuclease, and is presumably mobile. These genomes utilize an *attB* site for integration that has not been previously reported for mycobacteriophage prophage establishment, and we describe efficient Omega-derived integration vectors for transformation of both *M. smegmatis* and *M. tuberculosis* that are compatible with other integration vector systems.

## Materials and Methods

### Bacterial and Bacteriophage Strains

The isolation and genome sequences of Phages Omega, BAKA, Thibault, LittleE, Courthouse and Optimus have been described previously [1,6]. All infect and were propagated on *Mycobacterium smegmatis* mc^2^155 [18]. High titer lysates were generated on solid media, and harvested lysates were filtered through a 0.2μ filter. The accession numbers for the phages are: BAKA [GenBank: JF937090], Courthouse [GenBank: JN698997], LittleE [GenBank: JF937101], Omega [GenBank: AY129338], Optimus [GenBank: JF957059], and Thibault [GenBank: JN201525]. No permits were required for the described study, which complied with all relevant regulations.

### Bioinformatic Analysis

Genome sequences were analyzed using DNA Master (http://cobamide2.bio.pitt.edu/). Genes were determined using Glimmer [19], GeneMark [20], Aragorn [21], and visual inspection. Functions were assigned where possible using BLASTP alignments to GenBank, or HHPred [22]. Comparative genomic analyses were performed with Phamerator [23] using database Mycobacteriophage_220. This program uses pairwise comparisons of gene products to sort genes into phamilies of related sequences (designated Pham 1, Pham 2, etc). Genome maps are generated from Phamerator in which the genes are color coded according to their Phamily, with orphams (those with only a single gene member) show in white [23].

### Protein composition of Omega and LittleE Virions

Omega and LittleE virions were concentrated by PEG precipitation and then purified by CsCl ultracentrifugation. Concentrated particles were mixed with CsCl (approximately 0.8 gm per ml of phage), and centrifuged at 38K rpm for 16hr at 18°C in a Ti 70.1 rotor in an ultracentrifuge. The visible band of phage particles was removed using a 3 ml syringe and 18-gauge needle. The phage band was dialyzed to remove CsCl. Purified particles were analyzed by SDS-PAGE by heating the sample with a buffer containing SDS and β-mercaptoenthanol prior to loading on a 12% polyacrylamide gel, and electrophoresed until the dye front ran off the bottom of the gel. Protein bands were visualized by staining with Coomassie blue. A second SDS-PAGE gel of Omega particles was prepared in which the sample was eletrophoresed only until the proteins reached the resolving gel. The single resulting band, comprised of all the particle proteins, was stained with Coomassie blue and excised from the gel. The protein were then eluted and digested with Trypsin. The tryptic peptides were analyzed by tandem mass-spectrometry (MS/MS) and their masses were compared to a library of putative proteins encoded by the Omega genome for identification by the University of Pittsburgh Genomics and Proteomics Core Laboratory (UPGPCL).

### N-terminal sequencing of Omega and LittleE capsid proteins

CsCl gradient purified Omega and LittleE particles were loaded into a 12% polyacrylamide gel containing SDS and electrophoresed until the sample dye ran off the bottom of the gel. The proteins were then transferred to PVDF membrane by electroblotting at 100v with CAPS buffer for 30min at 4°C. The membrane was then stained with Coomassie blue. Capsid bands, identifiable as the most intense band in each lane, were then subjected to multiple rounds of Edman degradation and HPLC analysis for amino acid identification.

### Cryo-electron Microscopy of BAKA

Purified samples of phage BAKA were prepared for negative-stain electron microscopy by placing 3 µl on a freshly glow-discharged carbon-coated copper grid, blotting, washing in 2% ammonium molybdate stain solution, blotting and air-drying. Grids were imaged in an FEI Tecnai T12 microscope (FEI, Hillsboro, Oregon) operated at 120 kV and a nominal magnification of 30,000x. Images were collected on a Gatan Ultrascan 1000 CCD camera (Gatan, Pleasonton, CA) with post-column magnification of 1.4x, and CCD elements of 14 µm, resulting in an effective pixel size of 3.3Å. For cryo-EM, 3 µl of sample was placed on a Quantifoil R2/1 holey-carbon grid (Quantifoil MicroTools, Jena, Germany), and blotted and plunge-frozen into liquid ethane using an FEI Vitrobot. Grids were transferred to a Gatan 626 cryo-holder and imaged in an FEI TF20 microscope operating at 200 kV and nominal 50,000x magnification using low-dose conditions and maintaining liquid nitrogen temperatures throughout. Images were collected on a Gatan Ultrascan 4000 CCD camera with post-column magnification of 1.4x, and CCD elements of 15 µm, resulting in an effective pixel size of 2.1Å. Capsid images were boxed out with the *x3dpreprocess* software [24], defocus estimates made manually with the BSOFT package [25], and image reconstructions were calculated using the AUTO3DEM software suite [26,27]. Reconstruction were visualized with the UCSF Chimera software [28]. 370 phage capsid images were collected from 31 CCD images, and 250 were used to generate the final density map, which had a resolution estimated at 22 Å according to the spatial frequency at which the Fourier shell correlation [29] calculated between half-dataset density maps dropped below a value of 0.5.

### RNA isolation and RT-PCR

*M. smegmatis* mc^2^155 cells were grown in 7H9 medium plus albumin-dextrose-NaCl, 1mM CaCl_2_, 50 µg/ml carbenicillin, 10 µg/ml cycloheximide to an OD600 of 0.4 and infected with BAKA or LittleE at a multiplicity of infection of 1-5. Infected cells were incubated with shaking at 37°C for two hours and then aliquots were mixed with RNAprotect (Qiagen) and harvested by centrifugation. Uninfected cells were included as a control. Cell pellets were stored at -20°C prior to RNA extraction. RNA extraction was performed using the RNeasy mini kit (Qiagen) according to manufacturer’s instructions. RNA was treated with RNaseOut (Invitrogen) and then incubated with DNase I (Ambion) at 37°C for 1 hour. DNase I was then inactivated using DNase inactivation reagent (Ambion) The inactivation reagent was removed by centrifugation and the RNA was stored at -20°C prior to use as a template in gene specific reverse transcription. Primers were designed to amplify the transcripts for LittleE’s major capsid protein (GGTGTTATCGCCAGCCTGAACG) and for BAKA’s minor tail protein (GAATGTGACCTCGCCAGAAATATAGC) so as to include the putative intron splice site. Approximately 1 µg of RNA was incubated with 2 µl of a 10mM gene-specific primer stock in a total volume of 12.5 µl at 65°C for 5 mins and then chilled on ice. Reverse transcriptase, dNTPs and RTbuffer (Invitrogen) were added as according to instructions. The reaction was incubated at 42°C for 60min, and then at 70°C for 10 minutes to heat-kill the enzyme. Then the reaction was cooled to room temperature and incubated with RNase I (Qiagen) for 30min. Two µl of the cDNA was then used as a template in the final polymerase chain reaction amplification of the target genes. Primers used included the above primers along with CCTACGAGAAGGGCAACGG for LittleE and ACCTGCAGGAAGGGCTTTTCGGAGA for BAKA. PCR products were analysed by agarose gel electrophoresis for size and purity. The PCR product generated from LittleE infection was Sanger sequenced.

### Construction of Integration Vector pKR03

The Omega integration region (coordinates 53,200-55,040) was amplified by PCR using primers 5’-AAA AAA AAG CTT GAA GTC TTC GAG CGT CAT G and 5’-TTT TTT AAG CTT CAA GAT CCC TGG GGT TGA, digested with HindIII and cloned into similarly digested pMOS-Hyg, producing pKR03. Candidate integration-proficient vectors were verified by restriction digest and sequencing. For transformation efficiencies, 100 ng of pKR03 DNA was electroporated into either *M. smegmatis* mc^2^155 or *M. tuberculosis* mc^2^ 7000 competent cells, recovered and plated on selective media. Confirmation of *attB* integration sites was completed with PCR using a vector specific oligonucleotide and a nonspecific host oligonucleotide. PCR products were sequenced and the site of integration into the host chromosome was determined.

## Results and Discussion

### General features of phages Omega, BAKA, Courthouse, LittleE, Optimus and Thibault

Isolation and genome sequencing of the six phages discussed here has been described previously [1,6]. They come from geographically dispersed locations (Pennsylvania, North Carolina, Indiana, Massachusetts, Michigan) and all form turbid plaques approximately 3 mm in diameter when grown under standard conditions for 48 hours at 37°C (Table 1). All are dsDNA tailed phages with genomes ranging from 106,372 bp (Thibault) to 111,688 bp (BAKA) [1,6] (Table 1). The viral genomes are linear with 4bp 3’ single-stranded overhangs with the sequence 5’-ATCC, and non-homologous end joining is predicted to play a role in genome circularization following DNA injection [30].

**Table 1:** Features of Cluster J Mycobacteriophages.

**Phage**	**Length (bp)**	**GC%**	**ORFs**	**tRNAs**	**School**	**Location**	**GenBank**
BAKA	111688	60.7	245	1	Univ. Pittsburgh	Fort Bragg, NC	JF937090
Courthouse	110569	60.9	241	2	Indiana Univ. SE	Winchester, IN	JN698997
LittleE	109086	61.3	229	1	Lexington HS	Concord, MA	JF937101
Omega	110865	61.4	237	2	Univ. Pittsburgh	Upper St. Clair, PA	AY129338
Optimus	109270	60.8	230	1	Hope College	Holland, MI	JF957059
Thibault	106327	60.8	216	2	Bucknell Univ.	Lewisburg, PA	JN201525

### Cluster assignment

**Figure 1:**
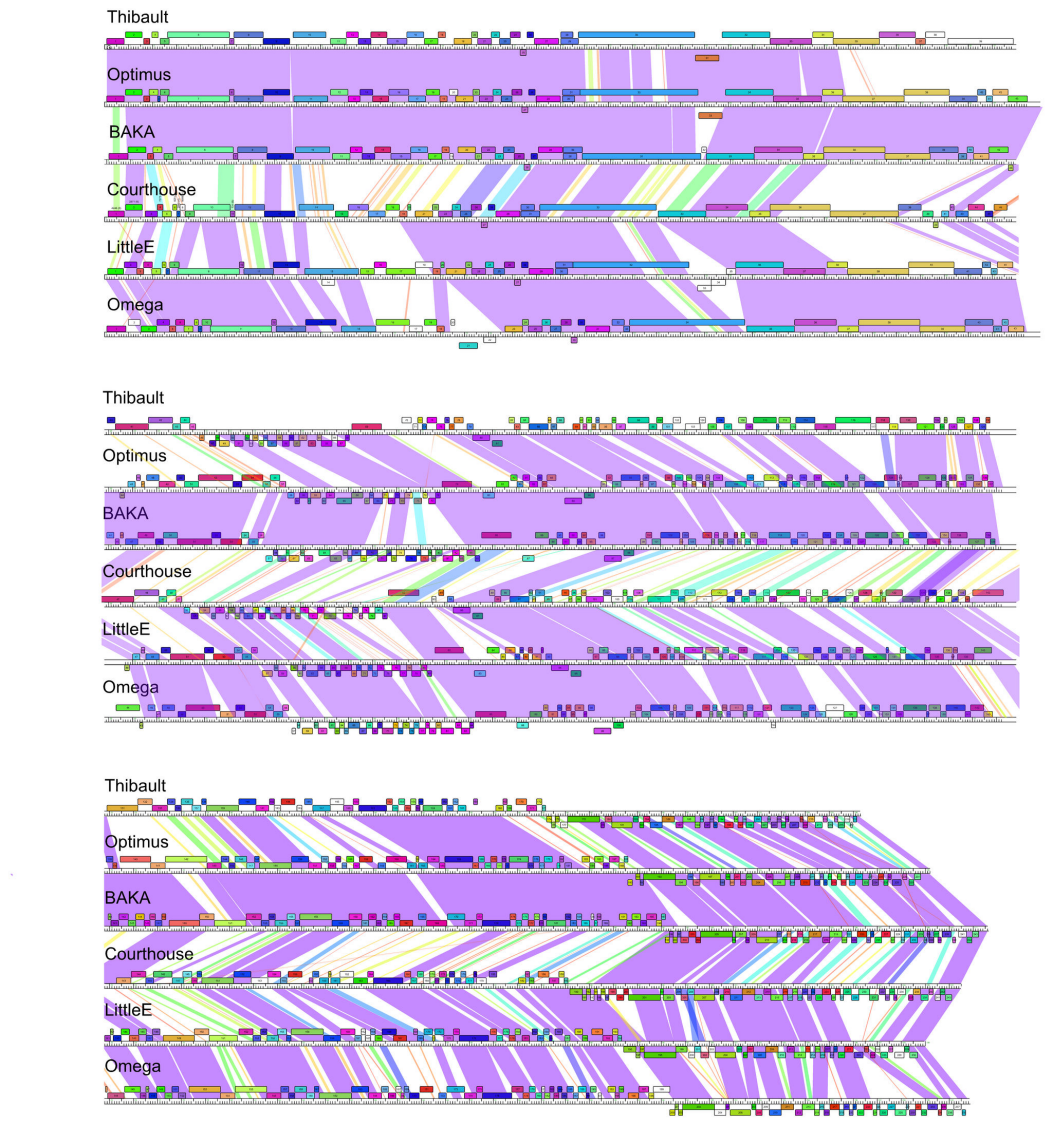
Comparative genomics of six Cluster J mycobacteriophages. Genome maps of six Cluster J phages, Thibault, Optimus, BAKA, Courthouse, LittleE and Omega are shown with genes represented as boxes above or below the genome, reflecting rightwards and leftwards transcription respectively. The color of each gene reflects membership in a particular phamily (see text for details). Coloring between genomes shows regions of nucleotide similarity determined by BlastN, with spectrum colors reflecting degrees of similarity: purple is most similar, red is least similar, and white shows no detectable similarity. Maps were generated using Phamerator, with database Mycobacteriophage_220 [23].

When phage Omega was first reported [6] it was one of 14 completely sequenced mycobacteriophages and represented a unique genome sequence with no close relatives. As the number of sequenced genomes increased to 30, those with nucleotide sequence similarity were organized into groups or clusters, and those with no close relatives, including Omega, designated as singletons [4]. Omega’s singleton status finally ended when the collection grew to 80 sequenced genomes [2], and further expansion to over 220 sequenced genomes [1] identified BAKA, Courthouse, LittleE, Optimus, and Thibault that share substantial nucleotide sequence similarity across their entire genome lengths with Omega; as such, these form the new Cluster J (Figure 1). The levels of average nucleotide sequence identity are reasonably high (Table 2), and the similarities extend across their genome spans, although there is considerable genome variation (Figure 1). The most distantly related genomes pairs BAKA/Courthouse and LittleE/Thibault (Figure 1 Table 2) have average nucleotide identities of 0.791, and these phages do not obviously warrant division into subclusters.

**Table 2:** Average Nucleotide Identities and Numbers of Shared Genes in Cluster J phages.

	**BAKA**	**Courthouse**	**Omega**	**Optimus**	**Thibault**	**LittleE**
**BAKA**	1/245	0.791	0.813	0.969	0.898	0.809
**Courthouse**	*173*	1/241	0.877	0.797	0.798	0.894
**Omega**	*169*	*172*	1/237	0.813	0.803	0.939
**Optimus**	*220*	*170*	*166*	1/230	0.902	0.811
**Thibault**	*167*	*157*	*154*	*165*	1/216	0.791
**LittleE**	*162*	*174*	*196*	*163*	*149*	1/229

Average Nucleotide Identities are shown in standard type. The numbers of shared genes – using standard threshold cutoff values of 32.5% identity or BlastP values of 10^-50^ or less – are shown in italic type.

**Figure 2:**
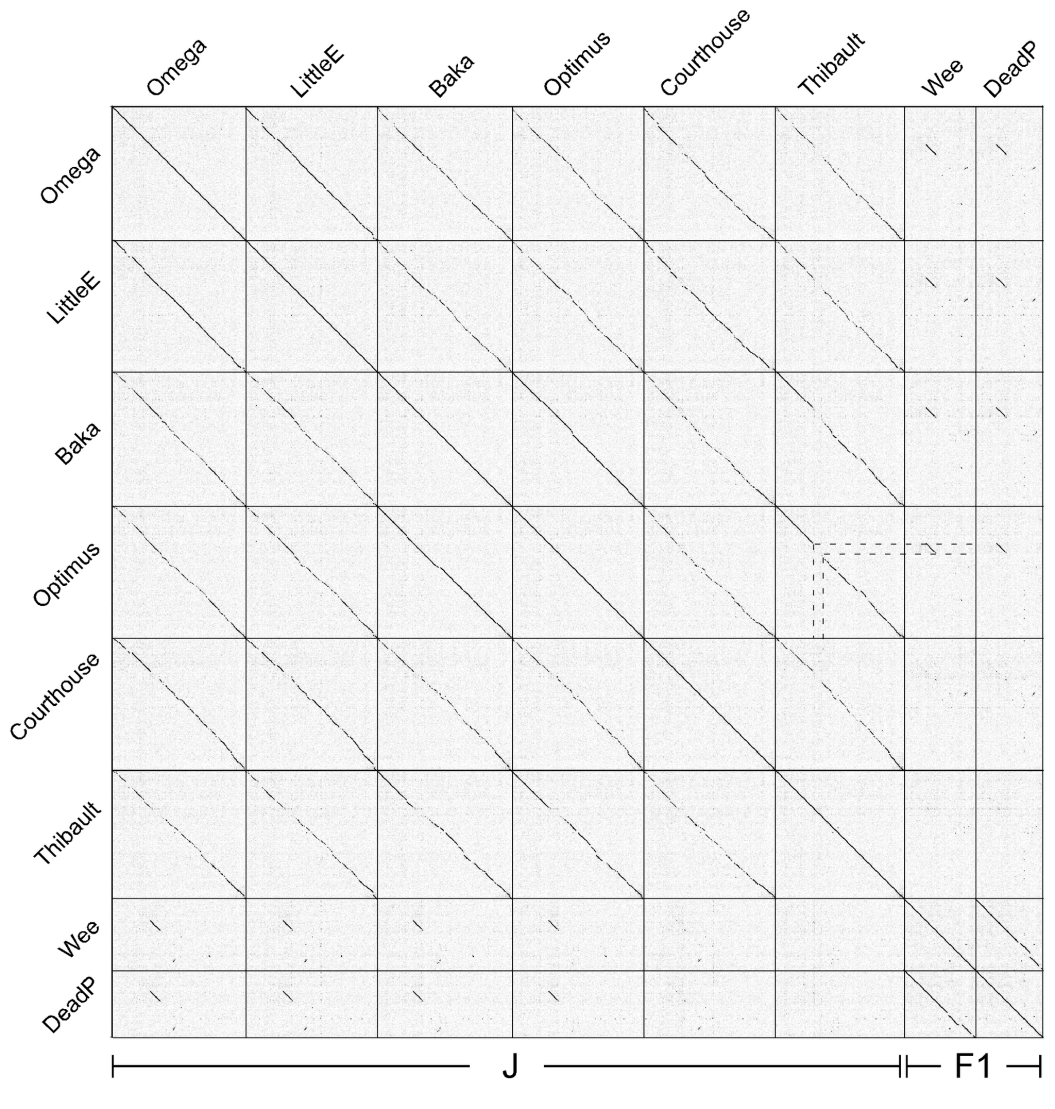
Dotplot of Cluster J and two Subcluster F1 phages. Whole genome nucleotide sequences of the Cluster J phages and phages Wee and DeadP from Cluster F1 were compared to themselves and to each other using the program Gepard [48]. Dotted lines are used to indicate the correspondence of the common segment in Wee and DeadP with the Thibault genome.

Typically, mycobacteriophage genomes within one cluster share little or no extended sequence similarity with other phage genomes. In a notable departure from this, some – but not all – of the Cluster J phages share a 6.8 kbp segment with many of the Subcluster F1 phages (with the exceptions of Shauna1 and ShiLan) at 94-96% nucleotide identity (Figure 2 Figure S1, S2). This region corresponds closely to a segment that differs in nucleotide similarity in Courthouse and Thibault from the other Cluster J genomes, and codes for minor tail proteins. In Omega, this region runs from bp 31,651-39,819, and includes genes 38-46 (Figure S2). Given the high level of nucleotide sequence identity, this shared region was presumably exchanged between Subcluster F1 and J phages relatively recently and it is plausible that these phages recognize a common host receptor.

### Cluster J phages are temperate and homoimmune

**Figure 3:**
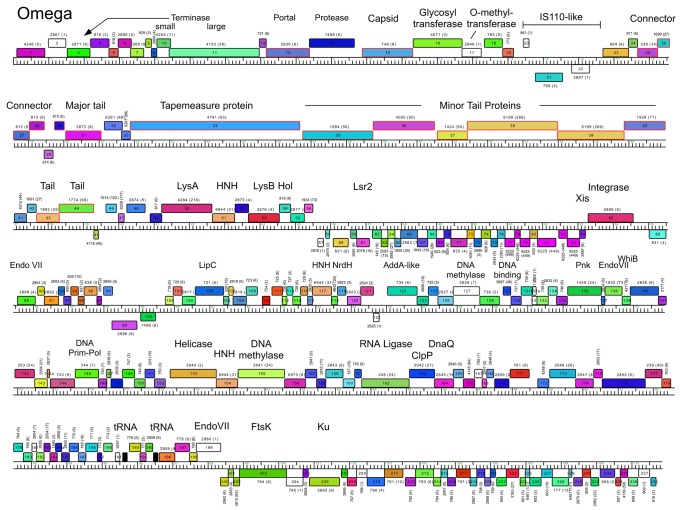
Genome map of Mycobacteriophage Omega. The Omega genome is shown with markers every 100 bp, and the predicted genes shown as colored boxes either above or below the genome reflecting rightwards and leftwards transcription respectively. Gene names are shown within the boxes, the corresponding Pham number is shown above or below each gene, with the number of Pham members shown in parentheses. Each gene is color coded according to its Pham assignment; genes designated as Orphams (i.e. in a phamily containing only a single member) are shown as white boxes. Predicted functions are shown above or below each gene. Genes whose products were identified as virion components are outlined in red. Maps were generated using Phamerator using database Mycobacteriophage_220 [23].

All of the Cluster J phages are temperate and form turbid plaques on *M. smegmatis* mc^2^155, from which stable lysogens can be recovered. Immunity tests show that these phages form a single immunity group and all Cluster J lysogens are immune to all other Cluster J phages (Figure S3). Consistent with this all of these phage genomes contain an integration cassette (see below), and all have a pair of divergently transcribed genes (e.g. Omega *100* and *102*), whose products have weakly predicted helix-turn-helix DNA binding motifs and are thus candidates for repressor and cro regulators (see Figure 3).

### Cluster J genome architectures

**Figure 4:**
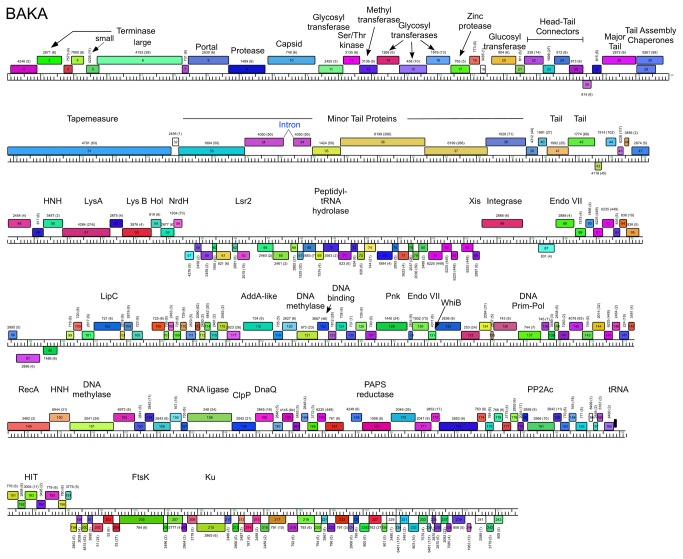
Genome maps of Mycobacteriophage BAKA. The annotated map of BAKA is represented as in Figure 3.

**Figure 5:**
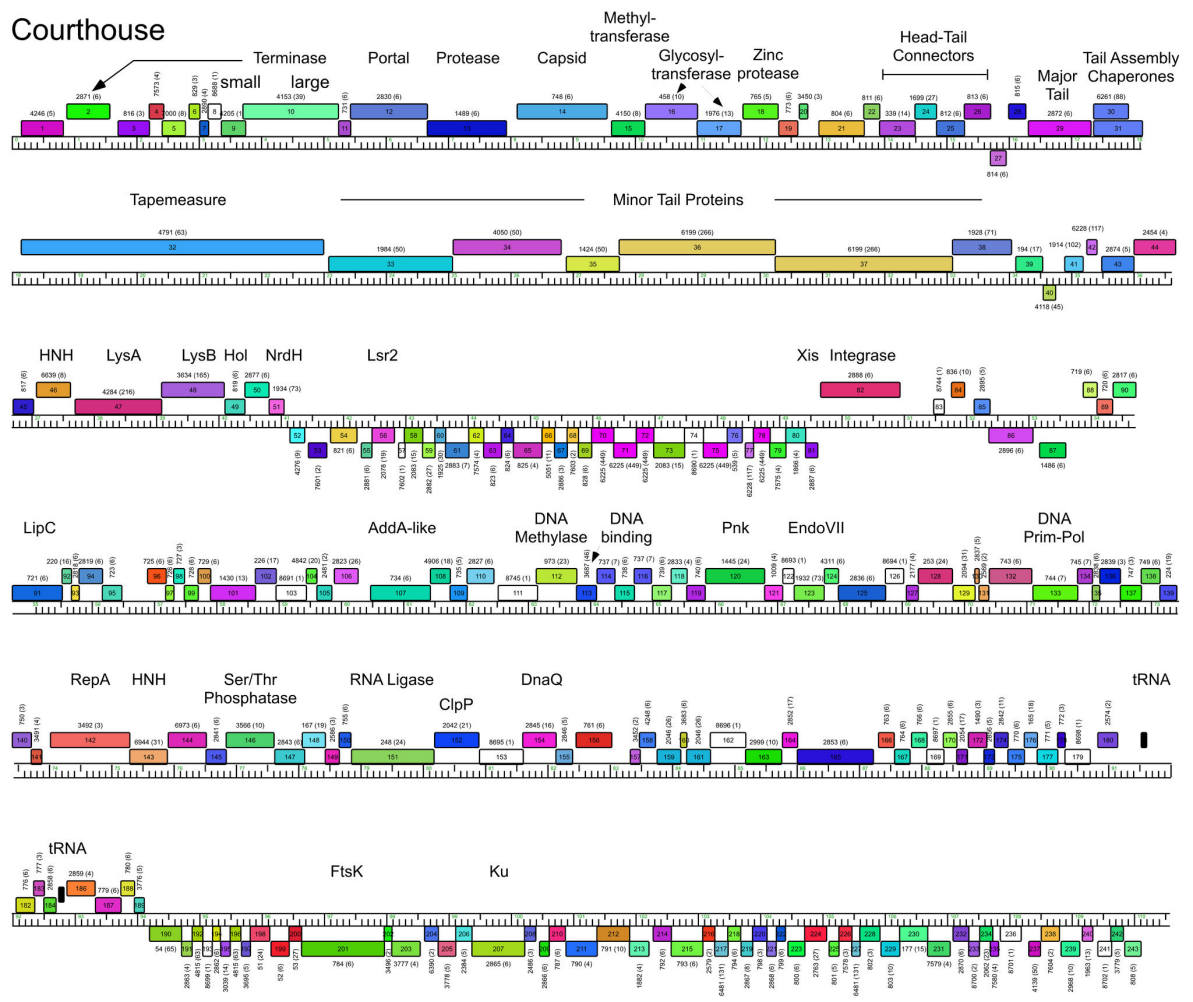
Genome maps of Mycobacteriophage Courthouse. The annotated map of Courthouse is represented as in Figure 3.

**Figure 6:**
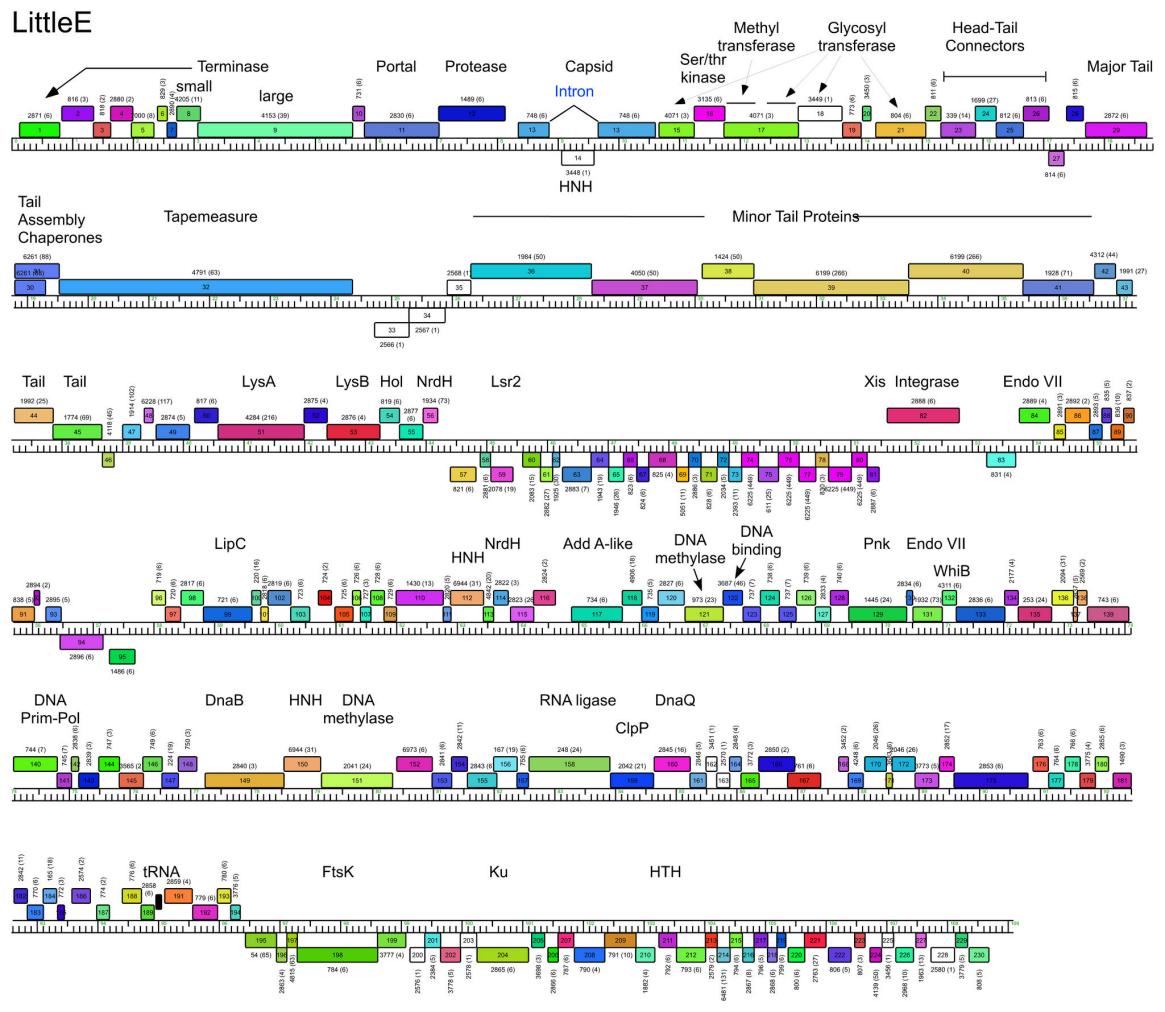
Genome maps of Mycobacteriophage LittleE. The annotated map of LittleE is represented as in Figure 3.

**Figure 7:**
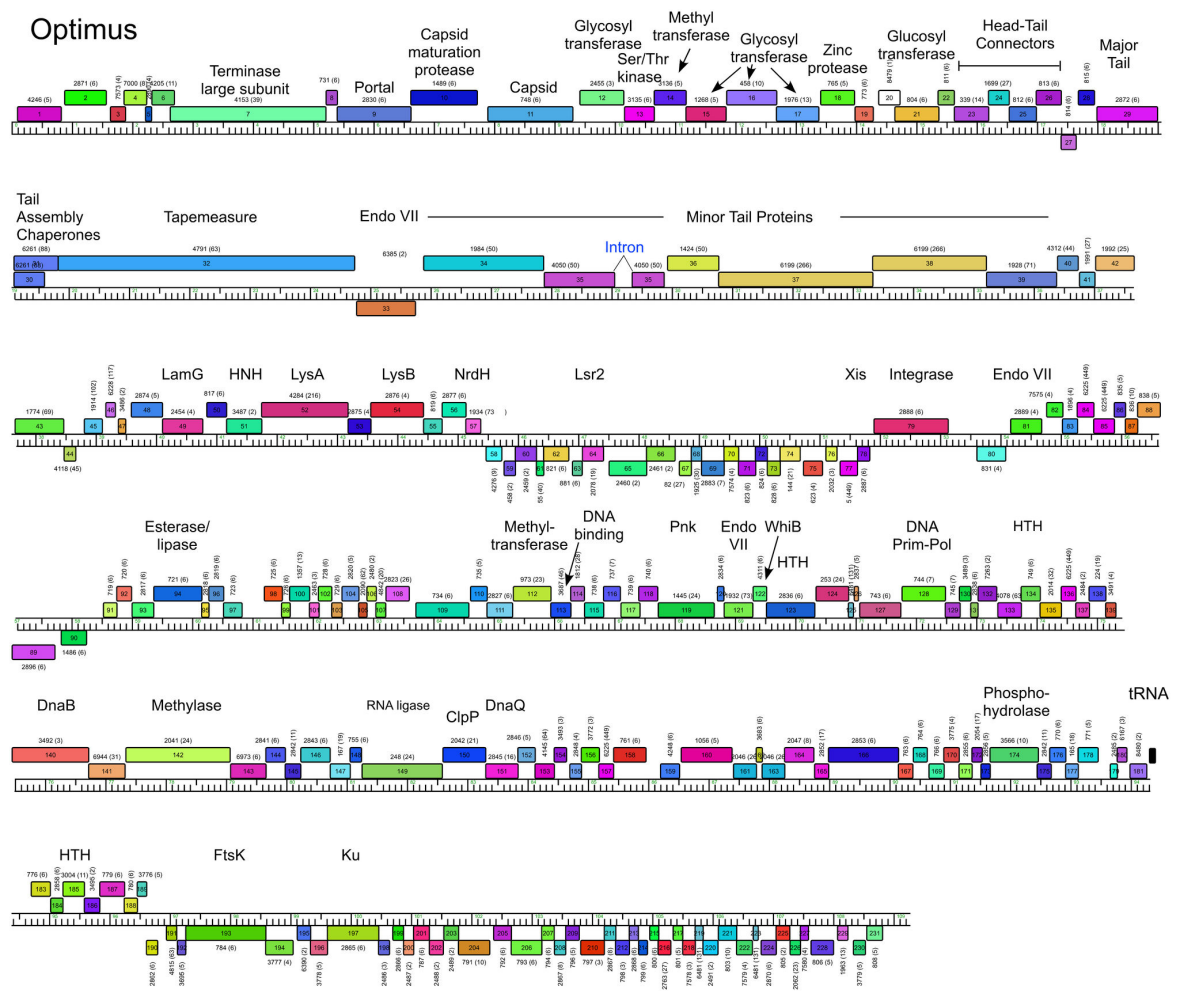
Genome maps of Mycobacteriophage Optimus. The annotated map of Optimus is represented as in Figure 3.

**Figure 8:**
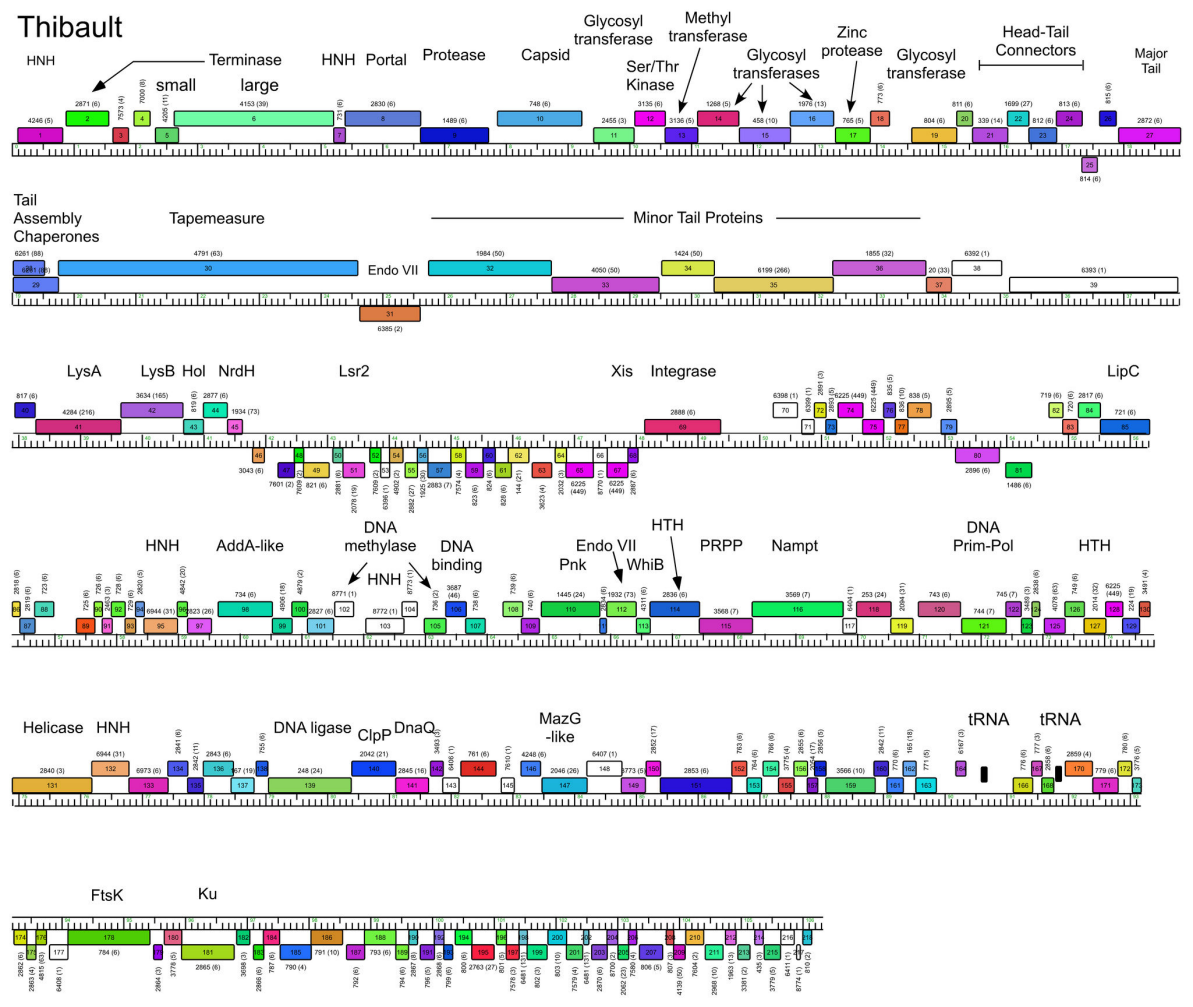
Genome maps of Mycobacteriophage Thibault. The annotated map of Thibault is represented as in Figure 3.

The six Cluster J genomes share similar architectures, with the integration cassette near the center of the genome (44.9–48% genome length from the left end) separating them into left and right arms (from left end to *attP* and *attP* to the right end respectively; Figures 3-8). The central location of the integration cassettes is a common feature among mycobacteriophage genomes [3,5], although it is somewhat surprising in these Cluster J genomes given that their overall genome lengths are 2-3 times greater than in most other mycobacteriophages with siphoviral morphotypes [1]. As such, the left arms not only contain virion structure and assembly genes occupying a total of ~ 25 kbp of genome space, but an equivalent portion of non-virion genes. The right arms are replete with small open reading frames of no known function, but also include genes involved in nucleic acid metabolism, RNA processing, tRNAs, and regulation. Detailed annotated maps are shown in Figures 3-8.

### Virion structure and assembly

**Figure 9:**
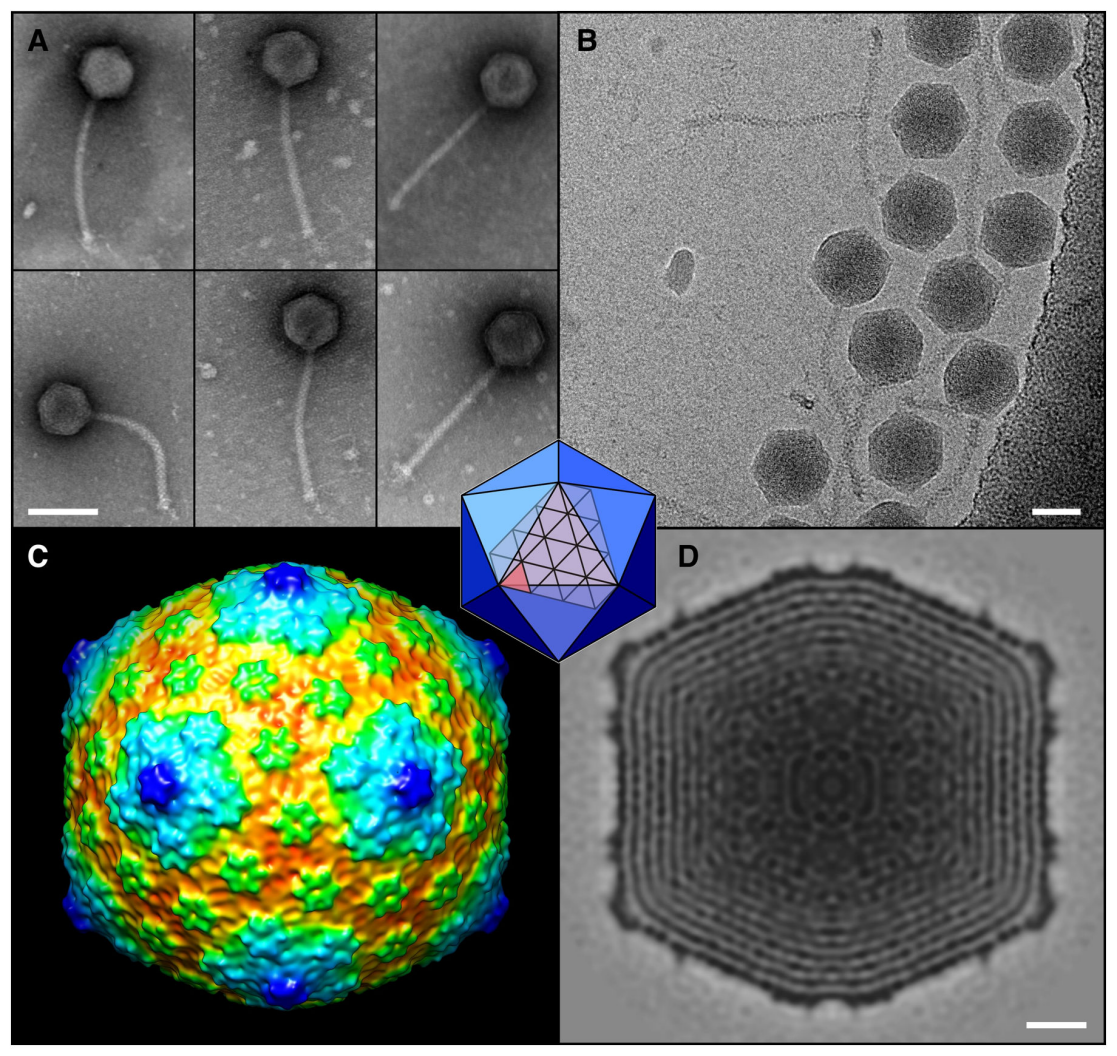
Morphologies of Cluster J phages. **A**. Electron micrographs of Cluster J phages. From left to right, top row: BAKA, Courthouse, LittleE; Bottom row: Omega, Optimus, Thibault. Phages were negatively stained with 1% uranyl acetate. Scale bar = 100nm. **B**. Phage BAKA Cryo-EM. The DNA-filled capsids are icosahedral with planar sides, and the long striated tail ends in a small two-layered base structure and tail tip. Capsids appear regular in the cryo-micrograph with small features evident at the outer surface and the tightly packaged dsDNA revealing the typical “fingerprints” of swirls and punctate patterns. Bar = 50nm. **C**, **D**. The capsid structure calculated from 250 particles to 2.2nm resolution is represented by surface shading colored by radius (C), or as a central thin section where dark regions correspond to the capsid (outer layer) and DNA (inner layers) spaced ~2.3nm apart (D). Capsomers on the vertices in panel C are at higher radii and appear dark blue, with adjacent hexavalent capsomers light blue and the remaining capsomers around the icosahedral 3-fold axes in green. The capsid diameter varies from 72nm across the 2-fold axis to 85nm across the 5-fold vertices. Organization of capsomers on the icosahedral lattice is described by a Triangulation number [49], T=13, in which the facet of the icosahedron is tiled by 13 smaller 3-subunit triangles (see inset). This organization is similar to that observed for coliphage T5 [31]. Bar = 10nm.

Electron microscopy (EM) shows that all six Cluster J phages have a siphoviral morphology with an isometric icosahedral capsid ~65 nm in diameter and a long flexible non-contractile tail approximately 200 nm in length (Figure 9). These phages have the largest capsid sizes and genome lengths among the flexible-tailed mycobacteriophages in the collection; the only larger ones belong to the Cluster C phages, which are all members of the *Myoviridae*. A cryo-EM reconstruction of the capsid of BAKA, the first for any of the mycobacteriophages, reveals an icosahedral lattice populated by pentameric capsomers at the vertices and hexameric capsomers on the facets arranged according to the triangulation number T=13 (Figure 9). Capsid diameter is 84 nM, slightly larger than measured by electron microscopy. Given the similar capsid dimensions and genome lengths, we predict that all the Cluster J phages likely share this capsid geometry. In morphology it closely resembles the coliphage T5 [31] and the isometric mutant of coliphage T4 [32,33], although it apparently does not include any surface decoration proteins. The tight layering of the genome, with spacing of the helices averaging ~2.3 nm, is typical of dsDNA tailed phages.

### Virion structure and assembly genes

**Figure 10:**
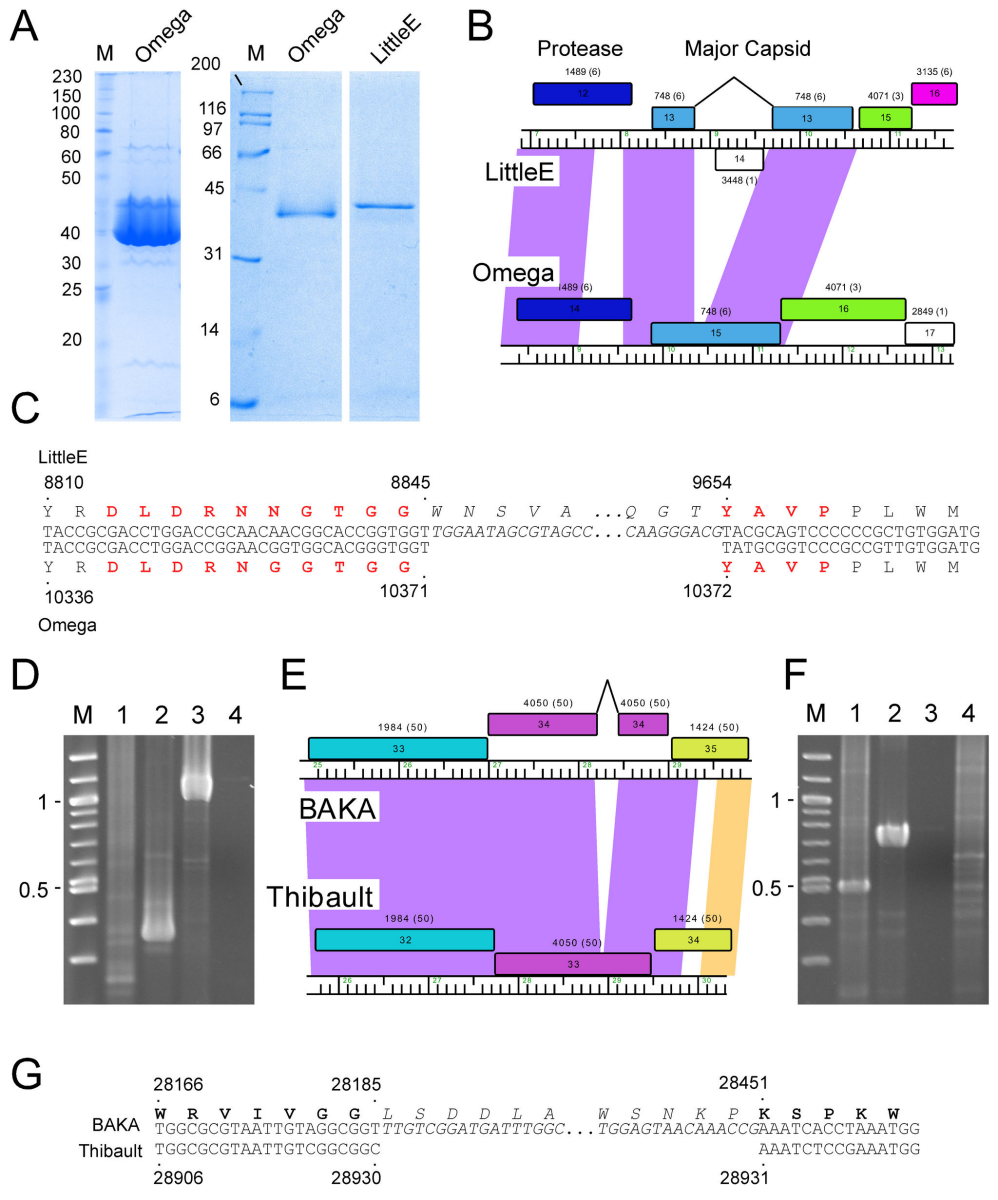
Intron splicing in LittleE and BAKA. **A**. 12% polyacrylamide-SDS gels of CsCl-purified Omega particles (left and center) and LittleE particles (right). In the left lane, Omega particles were heavily loaded to view less-abundant proteins. The major dark band corresponds to the major capsid as well as the major tail subunit protein that co-migrate. The lightly loaded sample in the right show the masses of the dominant protein species. **B**. Organizations of the capsid genes in Omega and LittleE. The major capsid genes are shown in blue, with the two exons of LittleE’s capsid gene connected by a black line. The HNH endonuclease (14) encoded within the LittleE intron is transcribed in the leftwards direction. Shading between the two genomes reflects nucleotide sequence similarity as in Figure 1. **C**. Nucleotide sequence alignment of the intron-splicing regions of LittleE and Omega. Amino acids translations are shown above the nucleotide sequence, with those determined by N-terminal sequencing in red. **D**. Agarose gel electrophoresis of RT-PCR amplification of the spliced LittleE intron. Lane M is a molecular weight ladder, with markers shown in kbp. cDNA amplification using LittleE primers used RNA from uninfected cells (lane 1), RNA from LittleE-infected cells (lane 2), LittleE phage genomic DNA (lane 3), and using RNA from infected cells but without reverse-transcriptase (lane 4). **E**. Map of minor tail protein regions of BAKA and Thibault, with the two exons of BAKA’s minor tail gene connected by a black line. **F**. Agarose gel electrophoresis of RT-PCR-amplification of BAKA intron region. Lane M is a molecular weight ladder, with markers shown in kbp. cDNA amplification using BAKA primers used RNA from BAKA-infected cells (lane 1), BAKA phage genomic DNA (lane 2), using RNA from infected cells but without reverse-transcriptase (lane 3), and RNA from uninfected cells (lane 4). **G**. Nucleotide sequence alignment of the spliced region in the minor tail gene in BAKA. Amino acid translations are shown above the nucleotide sequence, with exon-encoded residues in bold type.

In all six Cluster J genomes, the virion structure and assembly genes share the same synteny found in other phages with siphoviral morphologies, in the order: Terminase small subunit – Terminase large subunit – Portal – Protease – Capsid – Head-Tail Connectors – Major Tail Subunit – Tail assembly Chaperones –Tapemeasure Protein – Minor Tail Proteins (Figure 3). Assignment of virion gene functions is complicated by the interruptions in the left arms, but is facilitated by SDS-PAGE analysis (Figure 10A) and mass spectrometry of whole virions (Table 3). We will discuss these functions in the order they appear in the genome, starting with the terminases.

**Table 3:** Identification of Omega virion proteins by mass spectrometry.

Coverage(%)^1^	#PSMs^2^	# Peptides^3^	# AAs^4^	MW (KDa)^5^	Identity
37.11	138	11	477	51.5	gp15 Major Capsid
41.32	73	9	334	35.8	gp31 Major Tail Subunit
21.64	38	29	1599	167.2	gp34 Tape measure
30.56	35	17	661	72.8	gp35 Minor Tail
37.65	24	14	409	45.6	gp13 Portal
19.27	16	11	851	87.9	gp38 Minor Tail
37.81	18	7	283	29.1	gp37 Minor Tail
24.78	13	12	577	63.9	gp36 Minor tail
12.26	7	6	628	66.6	gp39 Minor Tail
23.81	7	4	189	20.8	gp25 head to tail
31.13	6	5	212	21.8	gp43
12.44	4	3	386	39.3	gp40
12.58	3	2	326	30.5	gp44
24.29	4	3	140	15.9	gp28 head-to-tail
16.78	3	2	149	17.3	gp27 head-to-tail
20.88	2	2	91	9.7	gp30 head-to-tail
7.89	1	1	114	12.6	gp26 head-to-tail

1 Coverage (%). Number of recovered amino acids compared to the predicted total number of amino acids in the protein.

2 Peptide spectrum matches (PSMs). Total number of peptide spectrum matched to predicted spectra based on the database generated from all proteins encoded in the annotated file for the Omega genome.

3 Unique peptides found

4 Total amino acids found within peptide spectrum matches

5 Predicted molecular weight of the likeliest whole gene product match

The terminase subunit genes are located near the left end of the genomes. The terminase large subunit gene can be confidently predicted bioinformatically because of similarities to other known terminases; apart from Courthouse gp10 all contain an intein (~340 aa) inserted around residue 104 (Figure S4). However, the relationships are complex. Omega gp11 is a close relative of LittleE gp9 (99% aa identity) including the intein, and both are closely related to the intein-less Courthouse gp10 (98% and 97% aa identity for extein 1 and extein 2 respectively). The inteins in Omega gp11 and LittleE gp9 are related to inteins (49% aa identity) in gp202 of the Cluster C phage ET08 and its relatives [2]. Thibault gp6, Optimus gp7 and BAKA gp6 are identical to each other, but more distantly related to Omega gp11 and its relatives (50% aa identity) although the second extein clearly is more closely related (71% aa identity) than either extein 1 or the intein. The genes immediately upstream of the terminase subunit (Omega gp10 and relatives) probably code for terminase small subunits (Figures 3-8), and have relatives in similar positions in distantly related genomes such as LeBron and relatives in Cluster L. However, Omega gp3, which has relatives in all other Cluster J genomes has weak similarity to the N-terminal half of terminase large subunits of a variety of other phages such as Wildcat (26% aa identity), Bongo (29% identity) and TM4 (25% identity). This arrangement is unusual and it is not clear if all three of the proteins (Omega gp3, gp10 and gp11, and relatives) play roles in DNA packaging.

The portal, protease and capsid subunit genes (Omega gp13, gp14 and gp15 and relatives; Figure 3) are all distantly related to proteins with similar functions in the well-studied HK97 [34]. The mature Omega gp15 capsid subunit is 478 residues long and shares a similar protein fold to the HK97 gp5 capsid subunit (Figure S5), including an N-terminal delta domain providing a scaffold-like function in capsid assembly. Confirmation of cleavage of the N-terminal delta domain in capsid maturation is provided by N-terminal sequencing of the LittleE gp13 capsid and the Omega gp15 capsid (see below), and cleavage occurs in an analogous position as in HK97 gp5 (Figure S5), although the Omega delta domain is somewhat larger (155 aa compared to 104 aa). The mature form of the capsid subunit contains several insertions relative to HK97 gp5, although predominantly at loops (with the exception of an extension of helix alpha5). Unlike HK97, the Omega capsid is not crosslinked, and alignment shows sequence variation at the critical residues required for crosslinking in HK97 gp5 (Figure S5).

It is common for the siphoviral mycobacteriophage genomes to have 4-8 structural genes between the capsid and the major tail subunits that provide the head-tail connector functions. The major tail subunit of Omega is clearly identified from mass spectrometry as gp31 (Table 3) but co-migrates with the processed head subunit by SDS-PAGE analysis (Figure 10A). Mass spectrometry identified gp25, gp26, gp27, gp28 and gp30 as Omega virion proteins and these are shared by all of the Cluster J phages; these are good candidates for head-tail connector proteins (Figure 3). Omega gp32 and gp33 correspond to tail assembly chaperones and are predicted to be expressed *via* a programmed translational frameshift and gp34 is the tape measure protein [6]. Omega gp35, gp36, gp37, gp38, gp39, gp40, gp43 and gp44 are all virion proteins by mass spectrometry (Table 3) and likely compose the tail tip structures. Omega gp39 (629 aa) is of interest in that it contains an N-terminal fibronectin type III motif (residues 3-66) and a β-lactamase-like D-ala-D-ala-carboxypeptidase motif (residues 64-268), a feature shared by many other unrelated phages, but which has not been shown previously to be a virion component. There is some variation of the tail proteins among the Cluster J phages with Thibault and Courthouse having the greatest differences (Figures 5, 8 Figure S2). Interestingly, Thibault gp36 also is predicted to have D-ala-D-ala-carboxypeptidase activity, but is not closely related (<30% aa identity) to those in the other Cluster J phages. Thibault gene 37 – for which homologues are absent in other Cluster J phages – is present in members of clusters A1, I1, O, as well as the singletons Marvin and Wildcat, and was determined to be part of the Marvin virion by mass-spectrometry [35]. The next two genes in Thibault, also likely minor tail proteins, are not present in any other mycobacteriophages, and this and the other variations in tail genes raises the possibility that this reflects the host preferences of these phages (Figure S2). 

### Intron splicing in Cluster J genomes

The major capsid subunit gene of LittleE (gp13) is divided into two distinct coding regions relative to its homologues in Omega and other Cluster J genomes (Figure 10B). Alignment of the DNA sequences indicates that LittleE has an insertion of 819 bp between coordinates 8,845 and 9,654, such that fusion of the left and right parts would generate a protein of the same length and similar sequence (90% aa identity) to Omega gene *11*. To test the hypothesis that the LittleE capsid gene is interrupted by an intron, we first examined the size of the LittleE capsid protein present in virions, which was found to co-migrate with the Omega capsid subunit (Figure 10A). We then determined the N-terminal sequence of the first 14 residues of the LittleE capsid subunit (N-DLDRNNGTGGYAVP), which begins at the site of processing (see above), and proceeds across an apparent splice site (Figure 10C). Finally, we confirmed that the processing event occurs at the RNA level by amplification of mRNA isolated from infected cells to generate a product 820 bp shorter than that from genomic DNA, and sequenced the product (Figure 10 C, D).

This intron is the first to be identified in any of the mycobacteriophage genomes. The capsid subunit is usually one of the most highly expressed proteins, and with a triangulation number of 13, each LittleE capsid is predicted to contain 775 copies of the capsid subunit. Presumably splicing is extremely efficient. The type of intron is unclear, but is most likely a group I. For example, the 3’ position of exon 1 is a U residue and the 3’ end of the intron is a G, both group I characteristics, and RNA folding suggests characteristics more similar to a group I than a group II intron [36] (Figure 10C). This intron is also predicted to be mobile as there is a reading frame on the bottom strand within the intron with similarity to a putative LAGLIDADG homing endonuclease of *Oedogoniumcardiacum* .

Interestingly, a DNA segment (156 bp) in the 5’ end of this LIttleE intron is 98% identical to a region in the tail genes of BAKA and Optimus (Figures 4, 6, 7). Specifically, BAKA (and Optimus) differ from Thibault (and the other Cluster J genomes) by an insertion of 265 bp that replaces the C-residue at Thibault coordinate 28,930 in gene 33 (Figure 10E). If this intervening segment were to be spliced out (removing BAKA coordinates 28,186–28,450), then the joined segments would code for a protein of the same length as Thibault gp33 (99% aa identity) and Omega gp36 (90% identity), a known structural protein (Table 3). RT-PCR analysis confirmed intron splicing (Figure 10F) and the spliced product deduced by comparison with the LittleE intron (Figure 10G) has a G at the 3’ end of the intron (genome coordinate 28,450) and a U at the 3’ end of exon 1 (genome coordinate 28,185). Unlike the LittleE intron, the BAKA intron lacks a homing endonuclease gene. 

### Non-structural gene interruptions in Cluster J left arms

The virion structure and assembly genes of the Cluster J phages contain an unusually large number of interruptions. At the extreme left ends of the genomes there are several genes to the left of the predicted small terminase subunit gene, and we note that Omega gp1 and gp7 (and their homologues) share some similarity (~65% aa identity over 97 aa) and HHPred analysis shows high probability matches (>88%) to a *Geobacter metallireducens* HNH endonuclease, phage T4 gp49 (Endo VII), and restriction endonucleases HPY99 I and PacI (Figure 3). Thibault gp31 and Optimus gp33 (each encoded immediately downstream of the tape measure protein) (Figures 7 & 8) are distantly related to these but have high probability scores to the same HHPred hits. Because none of these genes are closely linked to a DNA modifying enzyme such as a methylase, it seems unlikely that these enzymes have functions in restriction. All of the phages encode a putative Endo VII in the right arm (Omega gp138 and relatives; Figure 3), and we therefore propose that these genes encode free-standing homing endonucleases.

**Figure 11:**
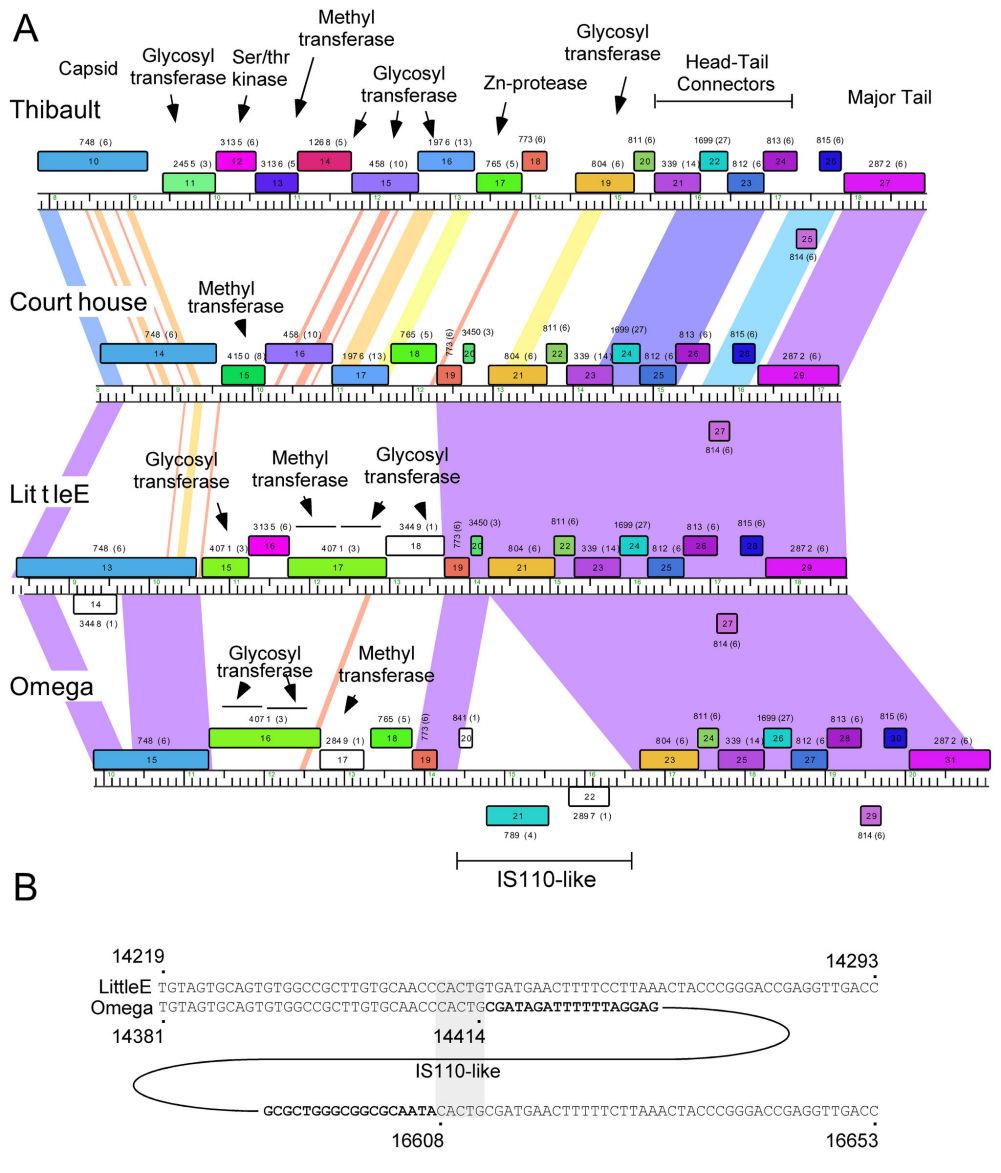
Interruptions between capsid and major tail subunit genes. **A**. Segments of the Thibault, Courthouse, LittleE and Omega genomes within the virion structural genes are shown, as described in Figures 1 and 3. These four genomes are diverse in this region with multiple different types of insertions, including an IS*110*-like transposon in Omega. BAKA and Optimus (not shown) are similar to Thibault in this region (see Figure 1). **B**. The IS*110*-like insertion region in Omega. Sequence alignment of the Little and Omega sequences reveals the limits of the transposon insertion in Omega (bold type) and five-base duplication of the target.

Another notable interruption is the insertion of 8-10 genes between the major capsid subunit and the head-tail connector genes (Figure 11A). This region is well-conserved in Thibault, Optimus and BAKA, with variations in Courthouse, LittleE and Omega (Figures 3-8). Although the specific roles of these genes are not known, many of them can be assigned functions. In particular, Thibault gp11, gp12, gp14, gp16, gp19, and their relatives in the other phages are all predicted to have glycosyl transferase activity, and have high probability HHPred matches (>99%) to a large series of structurally-determined glycosyl transferases (Figure 8). Omega gp16, LittleE gp15 and gp17 are also glycosyl transferases but have a complex relationship. Omega gp16 has two catalytic domains as in *Escherichia coli* chondroitin synthase [37], and LittleE gp15 and gp17 are related to each of these domains (54% and 56% aa identity respectively). However, the N-terminal half of LittleE gp17 is a predicted S-adenosylmethionine-dependent methyltransferase and is related to Thibault gp13, Optimus gp14, and BAKA gp13 (51% aa identity). Omega gp17 is also a predicted S-adenosylmethionine-dependent methyltransferase but has little similarity to LittleE gp17 or its relatives; the unrelated Courthouse gp15 also encodes a predicted methyltransferase (Figure 11A). LittleE gp16 (and its relatives in Thibault, Optimus and BAKA) encodes a putative serine–threonine kinase; it shares a short segment of similarity (64% identity over 47 aa) with Omega gp2. Finally, the 170-residue Omega gp18 and its homologues Courthouse gp18, BAKA gp17, Optimus gp18 and Thibault gp17 have unusual structures. HHPred reveals a high probability match (98%) of the C-terminal half of Omega gp18 (residues 93-162) to the zinc-binding component of the catalytic domain of the *Bacillus anthracis* Lethal Factor with conservation of all the metal coordinating residues [38]. The N-terminal half (residues 11-102) has a high probability match (95%) to the N-terminal half of an M48 family zinc-dependent peptidase of *Geobacter sulfurreducens* (PDB 3cqb_A). Given the complexity of these genes and the types of predicted activities, the questions as to whether their targets are viral or host derived and what the consequences are for phage growth are of some interest.

### Transposon insertion in the left arm of Omega

It has been noted previously that Omega gp21 has some similarity to transposases of the IS110 family [3], although the size and extent of a possible transposable element was not known. This assignment is based primarily on segments of similarity between Omega gp21 and other putative transposases including those in *Mycobacteria*, *Rhodococcus* , and *Gordonia* . Omega, LittleE and Courthouse are closely related in this genome region, but LittleE and Courthouse lack the apparent transposon insertion in Omega (Figure 11B). Alignment shows a precise insertion in Omega generating a 5 bp (5’-CACTG) target duplication (Figure 11B). Other members of the IS110 family of elements are reported to not generate direct repeats of the target, so this element may represent a separate subclass of this family [39,40]. There are no inverted repeats at the ends of the Omega transposon, a feature of other IS110 family elements. Although some Subcluster A1 phages such as Bxb1, BPBiebs, and *Euphoria* encode distantly related homologues of Omega gp21 (51% aa identity), there are no other closely-related copies of this putative element in mycobacteriophages or elsewhere.

### Lysis cassette

All of the Cluster J genomes have a lysis cassette following the virion structural genes and transcribed rightwards (Figure 3 Figure S2). Each contains three genes coding for an endolysin (Lysin A), a mycolylarabinogalactan esterase (Lysin B) and a Holin. However, there is variation in these genes and their organization. For example, the endolysins of Courthouse (gp47) and Thibault (gp41) have different domain organizations [Org-A [41]] than Omega (gp50), BAKA (gp51), Optimus (gp52) and LittleE (gp51) [Org-N [41]]. Similarly, the Lysin B proteins of Thibault and Courthouse are also distantly related to those in the other four Cluster J phages (<30% aa identity), although they are all predicted to be mycolylarabinogalactan esterases. In all six genomes the putative holin is encoded immediately downstream of *lysB* and has two predicted transmembrane domains. In Thibault and Courthouse, *lysA* and *lysB* genes are adjacent to each other (Figures 5, 8), but the other four genomes have 1-2 intervening orfs. One of these orfs is shared by all four phages, and is of no known function. The additional insertion in Omega (gene *51*) codes for a HNH endonuclease, and is discussed further below.

### Integration cassette

**Figure 12:**
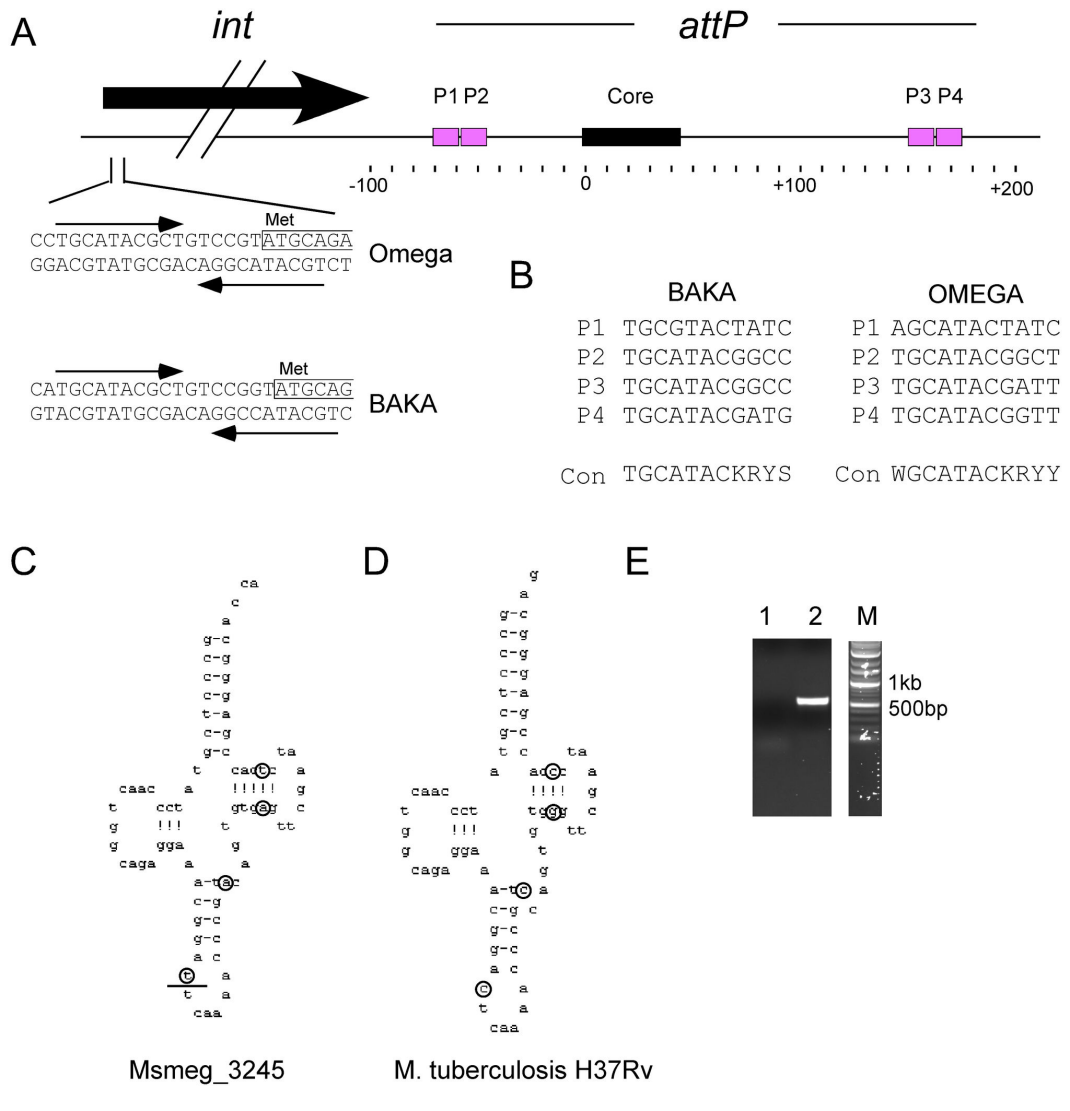
Integration functions of Cluster J phages. **A**. Architecture of the integration cassette in Cluster J phages. The *attP* site is located downstream of the integrase gene with the 44 bp common core – which is shared by the *attB* site – located approximately 100bp from the 3’ end of *int*. The common core is flanked by pairs of putative arm-type integrase binding sites, P1 and P2 to its left, and P3 and P4 to its right. At the start of the *int* genes of Omega and BAKA there two arm-type-like binding sites in inverted orientation overlapping the putative integrase start codons. **B**. Sequences of the putative arm-type binding sites in BAKA and Omega, with the consensus sequences (con). **C**, **D**. Schematic representations of the tRNA-Leu genes overlapping the common core at *attB* in *M. smegmatis* (C) and *M. tuberculosis* (D). Sequence differences between the two tRNAs are circled. The left end of *attB* site is indicated by a horizontal line in the anticodon loop of the Msmeg_3245 tRNA. **E**. Agarose gel of PCR products demonstrating integration of plasmid pKR03. Lane 1 is a control using pKR03 DNA and primers deigned to amplify *attR*. Lane 2 uses the same primers and DNA from a pKR03 transformant.

The integration cassette in the Cluster J genomes contains a rightwards-transcribed tyrosine-integrase gene and an *attP* site to its 3’ side (Figure 12A). The integrases are closely related, although the N-terminal arm-type binding domains of Omega, Courthouse and LittleE differ from those in the integrases of Thibault, BAKA and Optimus. Comparison of the phage genomes with *M. smegmatis* identifies a 44 bp sequence of identity located downstream of *int* (Omega coordinates 54,667–54,710) corresponding to the *attP* common core and this is identical in all six phage genomes indicating that they all integrate into the same *attB* chromosomal site at a tRNA-Leu gene (Msmeg_3245) (Figure 12C & D). Of all mycobacteriophages analyzed to date, the Cluster J phages are the only ones predicted to use this site for integration. The leftwards-transcribed genes immediately upstream of *int* (*84* in Omega) are strongly predicted to contain helix-turn-helix motifs and could act as recombination directionality factors [42].

Putative arm-type integrase binding sites flanking the *attP* common core are present as pairs of imperfect direct repeats 10 bp long related to a consensus sequence 5’-T**GC**A**TAC**g/tPuPy (positions conserved in all sites are in bold type) (Figure 12B). There are no obvious systematic differences in the sites that would correspond to differences in the N-terminal arm-type binding domains of the integrases. Interestingly, all of the phages contain an inverted repeat of related sequences at the start site of the integrase gene (Figure 12A). It is not known if the integrases can bind to sites in this inverted orientation, but it raises the possibility of a regulatory role of integrase in its own expression.

Because the Cluster J phages are the only ones predicted to use this particular *attB* site, we constructed an integration-proficient vector that should be compatible with other mycobacterial vectors. Plasmid pKR03 contains a DNA segment of Omega (coordinates 53,200-55,040) including *int* and the *attP* site, a hygromycin resistance marker, and an origin of replication for *E. coli*. Plasmid pKR03 lacks an origin of replication for *M. smegmatis*, such that when introduced into *M. smegmatis*, hygromycin resistant transformants arise by integrase-mediated insertion into the *M. smegmatis attB* site used by Omega. When pKR03 was electroporated into *M. smegmatis*, transformants were recovered at a frequency similar to that of a control plasmid, pMH94 containing the integration system of L5 [43] (~10^5^ transformants/µg) and PCR analysis showed integration into the predicted *attB* site, a tRNA-Leu gene (Figure 12E). Plasmid pKR03 similarly transformed *M. tuberculosis* mc^2^ 7000, however transformants were recovered at approximately an order of magnitude lower than that of the control plasmid.

### Non-structural genes

Because of the relatively large size of the Cluster J genomes, there are many genes (>200) involved in functions other than virion structure, lysis, or integration. In Omega these are organized into four leftwards-transcribed regions: 1) between the lysis and integration cassettes (~7.5 kbp), 2) a single gene (86) to the right of integrase, genes 99-100, and a ~12.5 kbp operon extending from the right genome end, 3) short (gene 89-98) and 4) longer (~40 kbp; genes 102–199) rightwards-transcribed operons (Figure 3). The other Cluster J genomes are organized similarly with minor variations (Figure 3). These regions are replete with small open reading frames, and only about 10% of the genes have predicted functions. Several of these warrant further discussion.

First, the presence of an FtsK-like protein in Omega (gene *203*) has been noted previously [6] and homologues are present in all of the Cluster J genomes but in no other mycobacteriophages. These contain an FtsK/SpoIIIE family conserved domain (pfam01580) and the closest relatives identified in a BLASTP search are the FtsK proteins of various mycobacteria (~60% aa identity within matching regions). However, the similarity only extends across the central FtsK motor domain (Figure S6). The N-terminal membrane-associated domain is missing in the phage genes, and the C-terminal ~90-residue DNA-binding γ-domains are present, but distinctly different in sequence. The function of the phage proteins is unclear, but we presume that they are binding and translocating along phage DNA. We cannot exclude the possibility that they recognize a host DNA sequence, although the γ-domains do not correspond to any of the more than two-dozen sequenced mycobacterial (or any other) bacterial homologues. Furthermore, although the phage FtsK proteins are generally closely related, the greatest regions of difference are within the γ-domains where 20% of amino acid residues are varied relative to 4% over the rest of the sequences (Figure S6). However, any role of these proteins in phage growth remains unclear.

Omega gp136 is a bifunctional polynucleotide kinase (Pnk) protein containing both kinase and phosphatase domains and is proposed to play a role – together with the phage-encoded RNA ligase (Omega gp162) and the phage-encoded tRNAs – in RNA repair to counteract host viral evasion mechanisms (Figure 3) [44]. Supporting this is the observation that although the Pnk and RNA ligase are not closely linked, they appear to have co-evolved; both genes are found in only the Cluster J, Cluster E and Cluster L genomes, and all members of each of cluster contain both genes in addition to at least 1 tRNA gene.

The Cluster J genomes all encode a Ku-domain protein (e.g. Omega gp206; Figure 3), which are distant relatives of the Ku domain proteins encoded by the Cluster O genomes (e.g. Corndog gp87). The Cluster J Ku proteins differ though by virtue that their C-terminal ~30 aa are closely related to Lsr2 proteins, including those encoded elsewhere in the Cluster J genomes (e.g. Omega gp61; Figure 3). The functions of all of these proteins are unclear, although the Ku proteins have been implicated in facilitating NHEJ in genome circularization following infection [30]. Unfortunately, we have not been able to construct Cluster J phage mutants using the previously described BRED approach [45] because we have been unable to recover plaques following transfection of Omega DNA into electrocompetent *M. smegmatis*. A plausible explanation for this is that the Ku protein is packaged into phage capsids and must be co-infected with phage DNA for circularization and phage growth. We did not identify Ku in mass spectrometry of Omega particles, but cannot rule out that it was either present below the level of detection or modified such as to generate peptides with unrecognizable masses.

Thibault encodes both a putative phosphoribosyl pyrophosphatase (gp115) and a nicotinamide phosphoribosyl transferase (gp116) that are absent in the other Cluster J phages, but are found in the Cluster C2 phage Myrna, as well as several members of the Cluster L phages (Figure 8). These enzymes are implicated in nucleotide metabolism and NAD scavenging pathways, and appear to result from insertion between a gene with a HTH-DNA binding domain common to all six Cluster J phages, and a gene of unknown function. The other five Cluster J phages encode a fairly large putative DNA methylase slightly down or upstream from Thibault’s insertion, which is absent in Thibault.

### Free-standing HNH homing endonucleases

Throughout the six genomes there are numerous genes with predicted HNH endonuclease domains, and these fall into three main groups. The first contains the members of Phams 4246, 3687, and 3487 (5, 46, and 2 gene members respectively) and have domains related to the gp49 of phage T4. The second contains members of Phams 6639, 6944, and 8772 (8, 31, and 1 members respectively), and are related to an SPO1 protein. The third is the gp14 protein which is encoded in the LittleE capsid intron, has no close mycobacteriophage relatives, but is related to the LAGLIDADG-type nuclease, I-CreI [46]. The C-terminal ~50 residues of this 177 aa protein are, however, related to the C-termini of HNH proteins of Pham 6944. The functional basis of this relationship is not clear, but a similar relationship has been reported for other HNH endonucleases [47].

**Figure 13:**
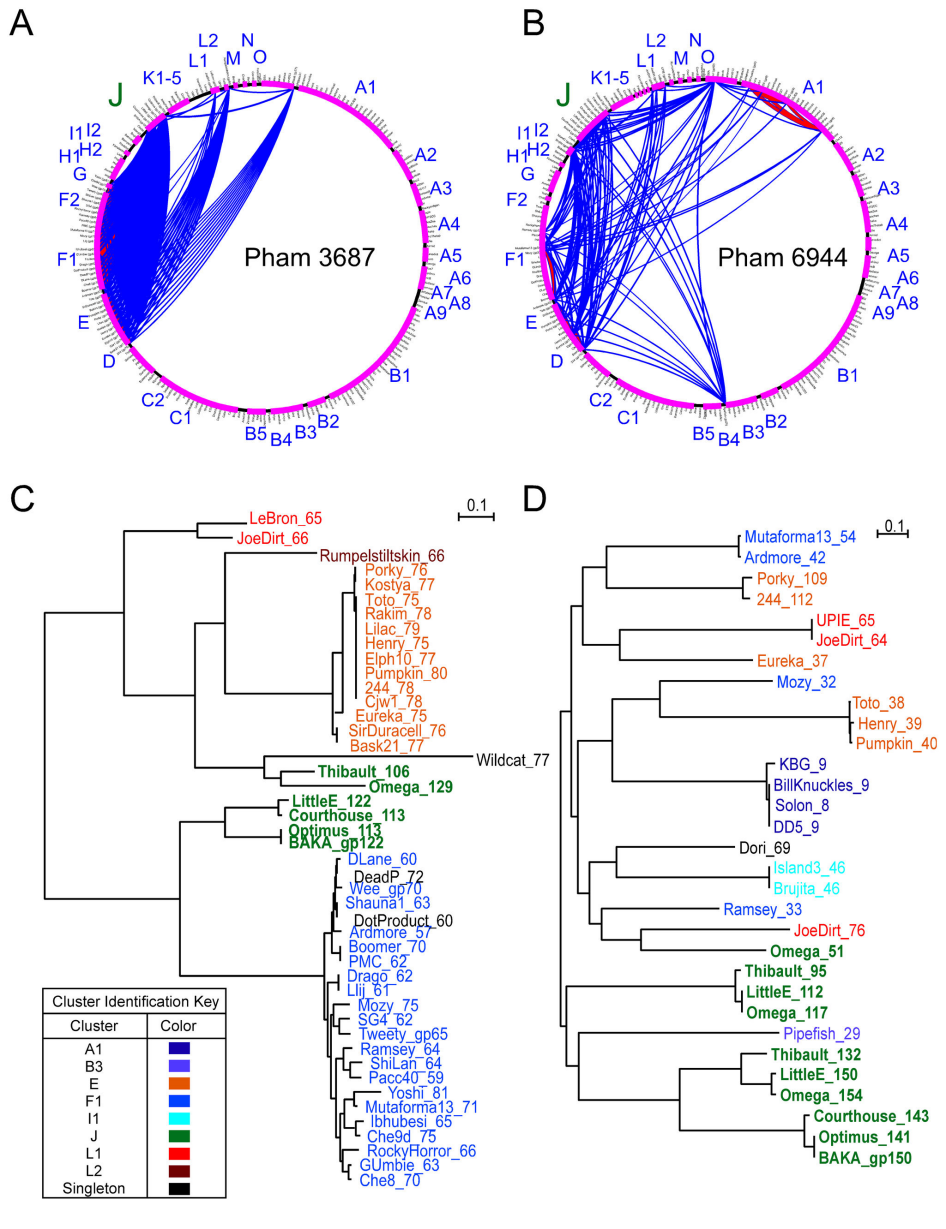
HNH endonuclease phamilies in Cluster J phages. **A**, **B**. Pham circles for phams 3687 (A) and 6944 (B). Phage names are organized by cluster/subcluster sequentially in a clockwise direction around the edge of the circle. Phages containing a gene within each pham are connected by an arc in blue (BLASTP) or red (ClustalW). Cirlces were drawn using Phamerator and thresholds of 32% identity and an E value of 10^-50^ for ClustalW and BlastP respectively. **C**, **D**. Phylogenetic trees of pham 3687 genes (C) and pham 6944 genes (D). Trees were generated using ClustalW multisequence alignments and neighbor-joining. Trees were drawn using NJPlot. Phages are color coded to designate cluster assignment, as shown in the key.

The distribution of members of the large HNH Phams 3687 and 6944 among the broader set of mycobacteriophage genomes is informative about their mobility (Figure 13 A & B). The 49 members of Pham 3687 are present in several different genome clusters (E, F1, F2, J, L1, L2 and Wildcat), but whenever it is present in one genome it is present in all genomes in that subcluster, and it is never present more than once per genome. Thus while it may be mobile, its rate of movement may be fairly modest. In contrast, Pham 6944 members are not only in several different clusters (A1, B3, E, F1, I1, J, L1 and Dori) but its presence varies considerably among phages within a subcluster, and it is often present more than once in the same genome (two copies in Thibault and LittleE, and three in Omega; Figures 3, 6, 8). It thus presumably moves at a somewhat higher rate. Phylogenetic trees also illustrate the mobilities of these HNH endonucleases (Figure 13 C & D).

**Figure 14:**
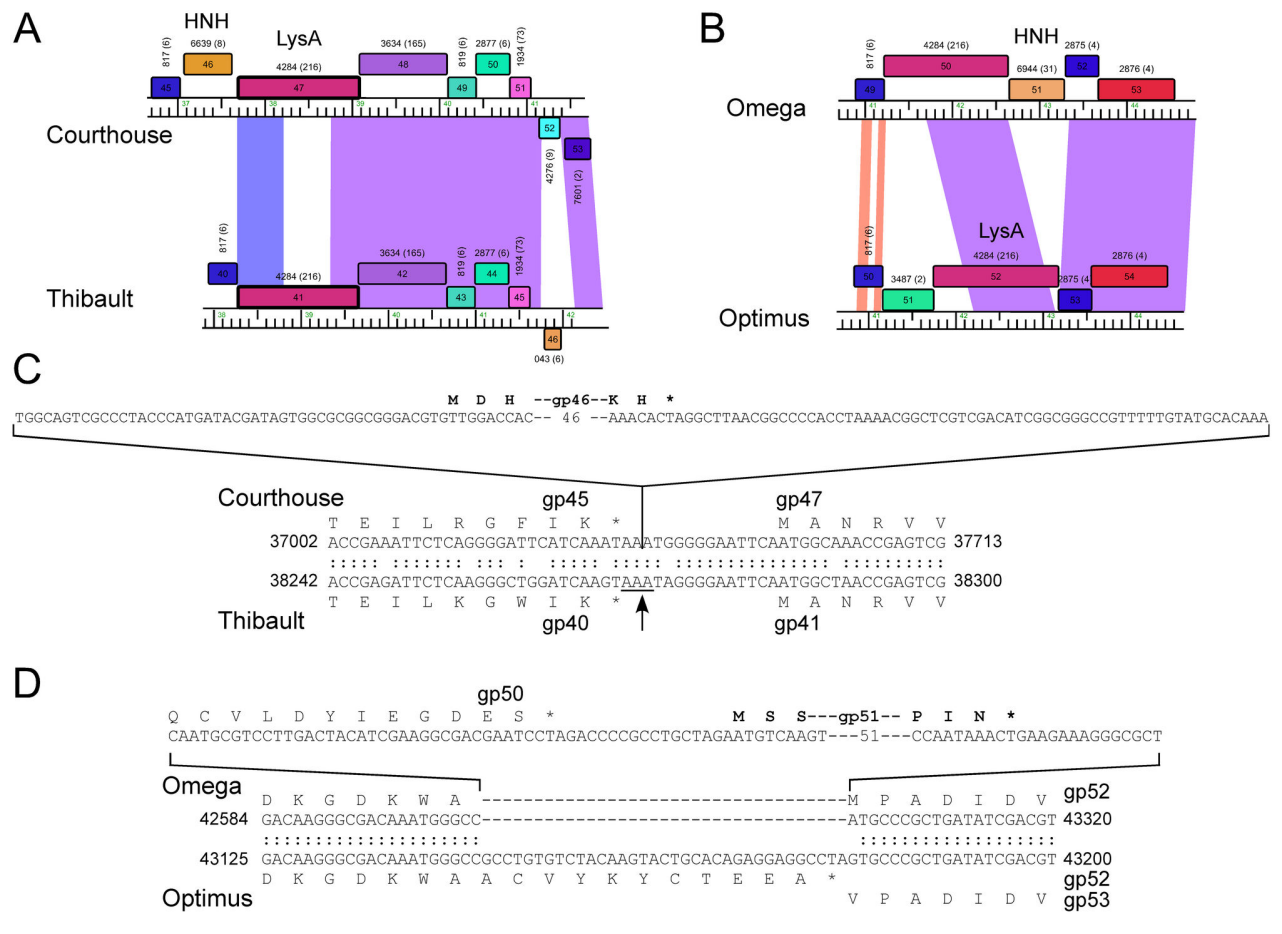
HNH endonuclease insertion regions. **A**. Regions including *lysA* genes showing insertion of a free-standing HNH endonuclease into Courthouse upstream of *lysA*. **B**. LysA region of Optimus and Omega, showing two different predicted HNH endonucleases, Optimus gp51 and Omega gp51. **C**. Sequence alignment of Courthouse and Thibault shows a precise insertion of predicted HNH endonuclease. **D**. Sequence alignment of Optimus and Omega, showing the HNH insertion site.

The presence of free-standing mobile HNH genes in otherwise closely related genomes provides opportunities to examine the likely sites of endonuclease recognition prior to insertion, and Courthouse gene *46* (Figure 14A) and Omega gene *51* (Figure 14B) represent two examples. In the first example, comparison of Courthouse and Thibault shows that the nucleotide sequences immediately surrounding Courthouse *46* are well conserved in Thibault (although it does not extend to the left through all of Courthouse *45* and Thibault *40*; Figure 14A), and the acquisition of Courthouse *46* can be accounted for by a precise insertion in the intergenic region corresponding to that between Thibault 40-41 (Figure 14C). Interestingly, the same location appears to have been used for insertion of an unrelated HNH endonuclease in phages BAKA and Optimus. In these examples, the left junction is precisely the same as that deduced from the comparison between Thibault and Courthouse (Figure 14A). However, the right junction is unclear as the 5’ ends of the adjacent Lysin A genes to the right have no close homologues elsewhere in the J genomes.

In the second example, the Omega gene *51* HNH endonuclease appears to have been inserted in a region corresponding to that between genes 52 and 53 of Optimus (and the identical regions in LittleE and BAKA; Figure 14B). However, the acquisition process is complex, as it is associated with loss of 35 bp from the presumed target sequence resulting in a new 3’ end of Omega lysin A (gene *50*; Figure 14D). Presumably, either errors were introduced during recombinational repair of the cleaved recipient, or the donating homing endonuclease was situated in a distantly related genomic context (either in another phage genome or perhaps elsewhere in the same genome). This illustrates that HNH endonuclease may play a significant role in generating genome mosaicism by creating rearrangement upon their movement. We also note that throughout the large set of mycobacteriophage sequenced genomes there are numerous examples (at least 18) of HNH insertions in or around the lysis cassette, raising the possibility that these endonucleases are implicated in contributing to the pervasive intragenic mosaicism that characterizes the lysin A genes [41].

### Concluding remarks

The comparative genomic analysis of the six Cluster J phages described here reveals them to be richly endowed with insights into phage genome structure, expression, and evolution. Their unusually large genome sizes among siphoviruses have many genes of unknown function, but also many genes whose functions can be predicted, but for which their specific roles in phage growth is far from clear. Identification of two introns was facilitated by access to a group of related phages, raising the question as to whether other – as yet unidentified - introns are present in other phages of high GC% Gram positive bacteria. Finally, the luxuriant mobilome of these genomes, with introns, inteins, transposons, and free-standing HNH endonucleases contributes to our understanding of phage evolution and the origins of mosaicism.

## Supporting Information

Figure S1 DotPlot of a large region of nucleotide similarity between Cluster J phage Optimus and Cluster F1 phage Wee. A portion of the Optimus genome is represented along the x-axis and a portion of the Wee genome is represented along the y-axis. Matching nucleotide sequence results in a diagonal line from top left to bottom right. Phamerator maps of the structural genes encoded by the regions are shown across the top and along the left sides of the plot.
(TIF)

Figure S2 Phamerator maps showing the region of the minor tail proteins in all the cluster J phages, as well as Cluster F1 phages Wee and DeadP. Four of the Cluster J phages share a region of nucleotide similarity with the cluster F1 phages: BAKA, LittleE, Optimus, and Omega. Courthouse minor tail proteins exhibit similarity at the amino acid level, and Thibault encodes some minor tail proteins unlike any other of the sequenced mycobacteriophages.
(PDF)

Figure S3 Homomimmunity of Cluster J phages. A high titer lysate of each of the Cluster J phages was serially diluted and spotted on *M. smegmatis* mc^2^155 (top left) and then on each of the mc^2^155 cluster J lysogens (labeled above each plate picture). Phage D29, a subcluster A2 phage, was included as a control.
(PDF)

Figure S4 Intein insertions in the terminase genes of the cluster J phages. Five of the six cluster J phages have an insertion near the N-terminus of their terminase proteins, but phage Courthouse does not. Genome maps show that the nucleotide identity of the start and end of the proteins is high. All five insertions appear to encode HNH endonucleases and are likely inteins. The five insertions are not identical; Omega and LittleE have one type of intein (red boxes), while Thibault, Optimus and BAKA have a different intein (light blue boxes).
(PDF)

Figure S5 HHPred alignment of the major capsid protein of Omega to the major capsid protein of HK97. Top row of the alignment is a secondary structure prediction, with upper case letters indicating more reliable predictions. The second line is the omega major capsid protein sequence and the third is the consensus hidden markov model prediction. The bottom rows are the subject rows, with the top one being the consensus hmm prediction, then the HK97 sequence, followed by two different secondary structure predictions. The central portion indicates areas of identity and similarity between the two hmm predictions. The black arrows indicate the site at which the protease cuts the major capsid proteins to remove the N-terminal delta domain from the mature capsid. The red arrow indicates the lysine residue responsible for the crosslinking of the mature major capsid proteins in HK97, which are absent from the Omega sequence.
(PDF)

Figure S6 Clustal alignment of the Cluster J FtsK proteins. The six proteins are highly conserved in sequence, with exception of the portion that likely corresponds to the DNA-binding gamma domain. These residues show more sequence variability, and do not match any known FtsK gamma sequences.
(TIF)

Table S1 Author Contributions.
(PDF)
