# Supplementary figures and images for: Cluster J Mycobacteriophages: Intron Splicing in Capsid and Tail Genes
Source: PLoS One. 2013 Jul 9;8(7):e69273. doi: 10.1371/journal.pone.0069273 (PMC3706429; doi:10.1371/journal.pone.0069273)

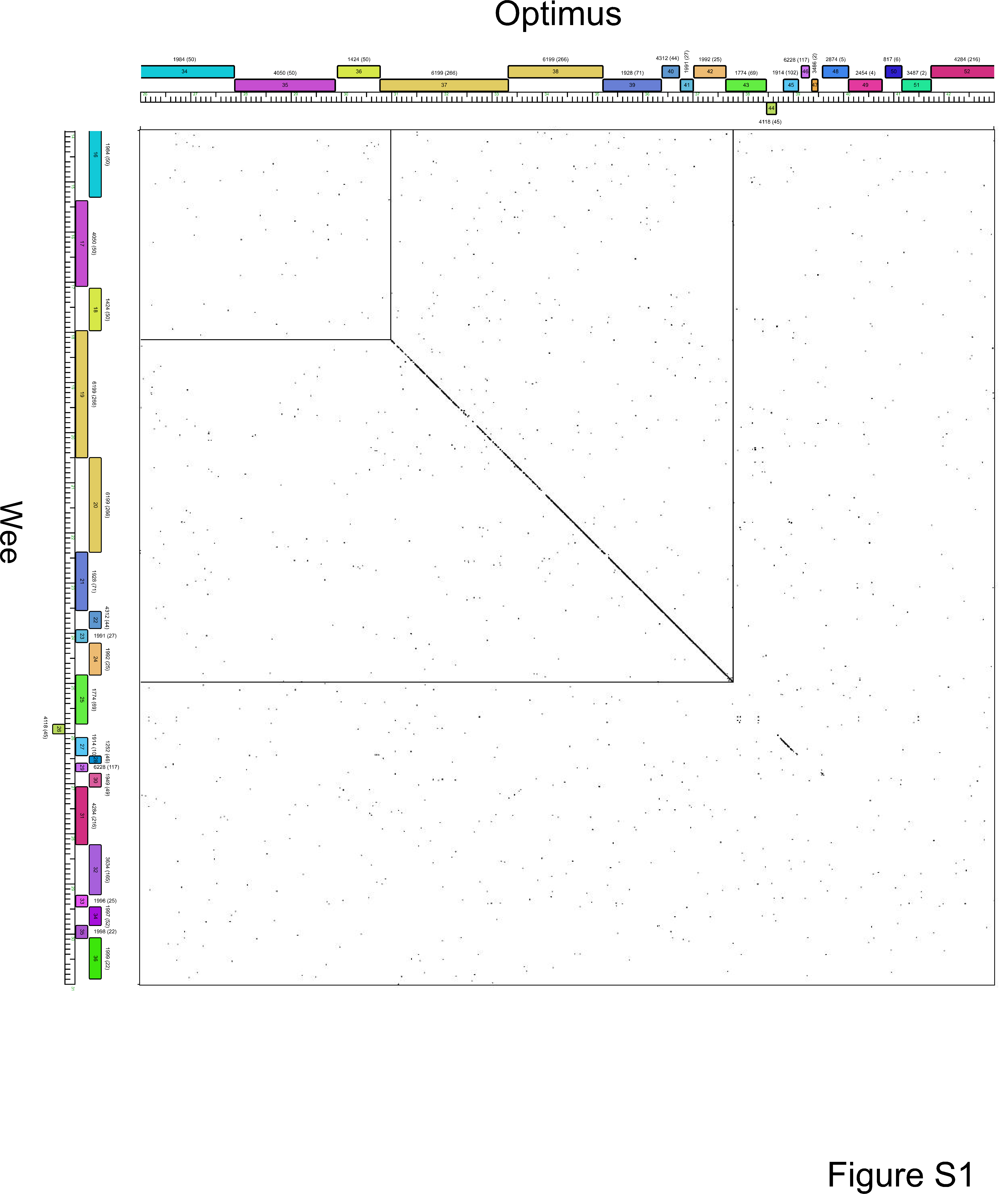

Supplement: Figure S1 — A portion of the Optimus genome is represented along the x-axis and a portion of the Wee genome is represented along the y-axis. Matching nucleotide sequence results in a diagonal line from top left to bottom right. Phamerator maps of the structural genes encoded by the regions are shown across the top and along the left sides of the plot. (TIF) [file pone.0069273.s001.tif]

# Courthouse

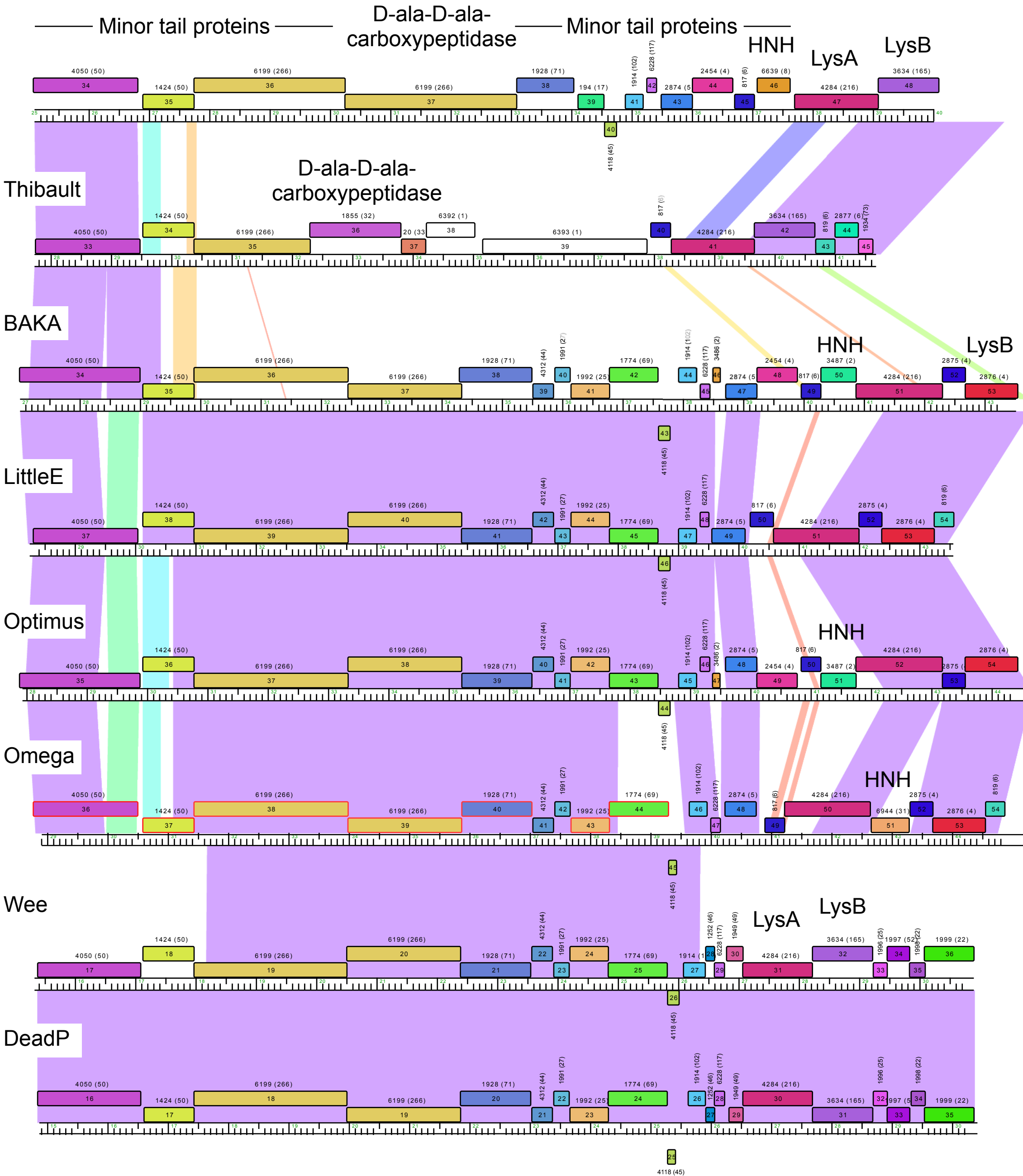

## Figure S2

Supplement: Figure S2 — Four of the Cluster J phages share a region of nucleotide similarity with the cluster F1 phages: BAKA, LittleE, Optimus, and Omega. Courthouse minor tail proteins exhibit similarity at the amino acid level, and Thibault encodes some minor tail proteins unlike any other of the sequenced mycobacteriophages. (PDF) [file pone.0069273.s002.pdf]

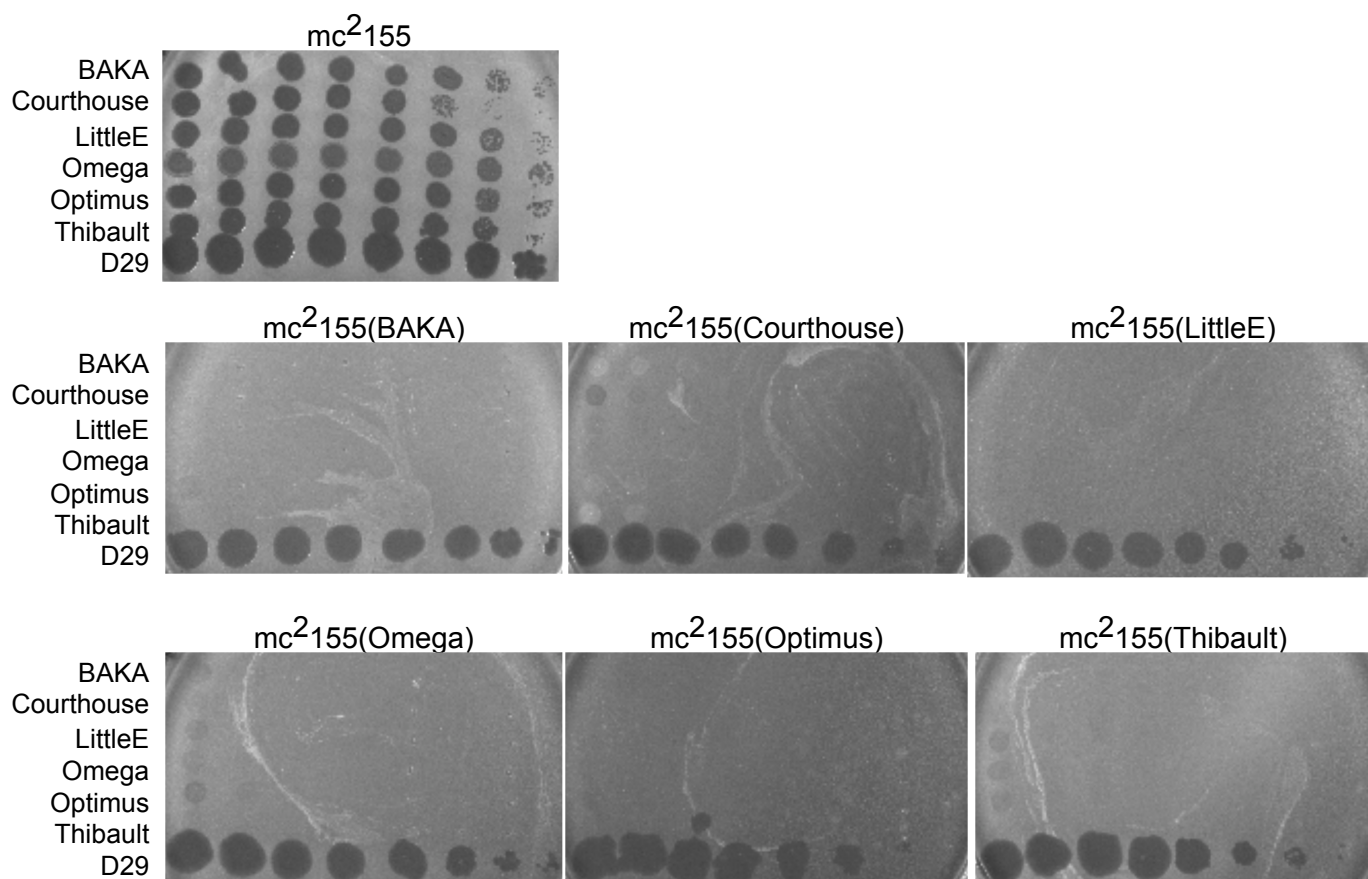

Figure S3

Supplement: Figure S3 — A high titer lysate of each of the Cluster J phages was serially diluted and spotted on M. smegmatis mc2155 (top left) and then on each of the mc2155 cluster J lysogens (labeled above each plate picture). Phage D29, a subcluster A2 phage, was included as a control. (PDF) [file pone.0069273.s003.pdf]

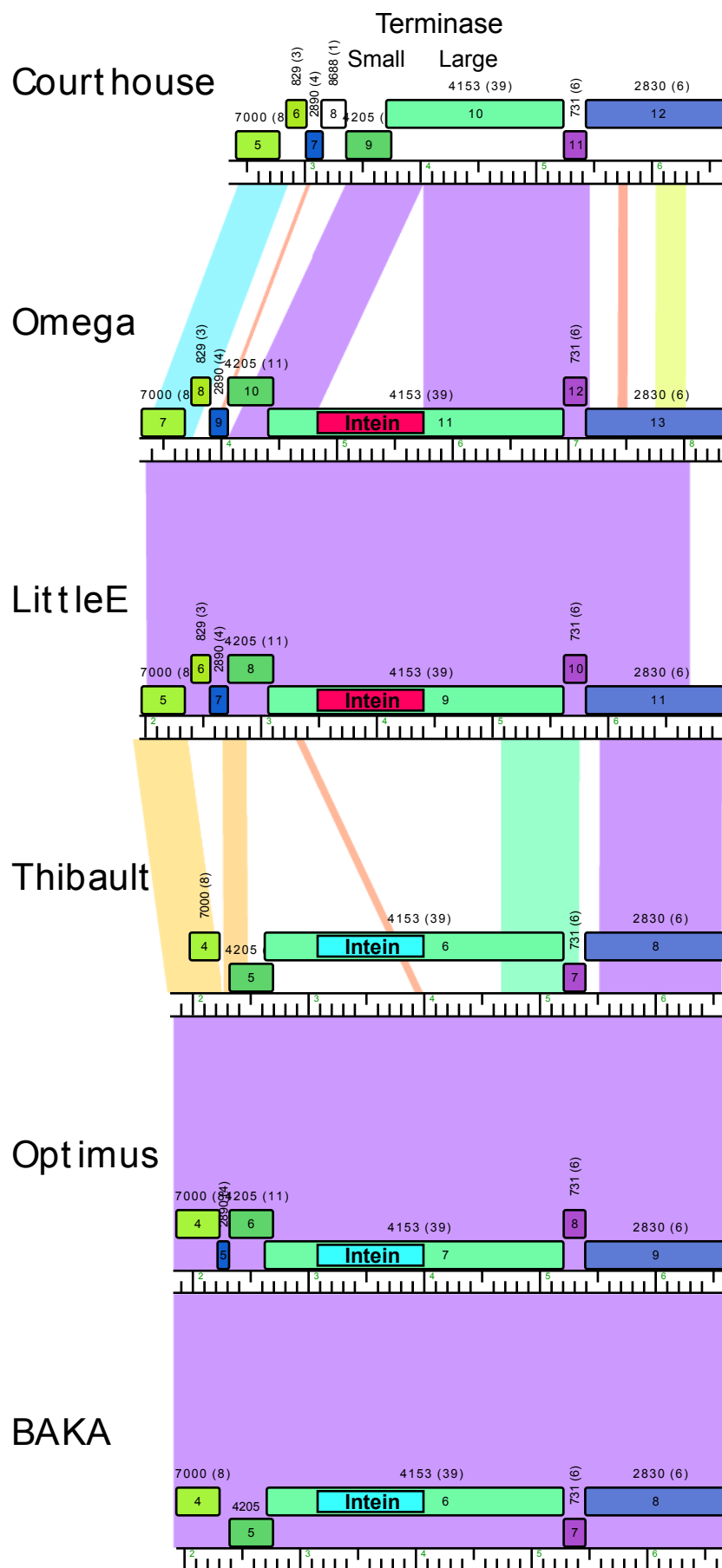

Figure S4

Supplement: Figure S4 — Five of the six cluster J phages have an insertion near the N-terminus of their terminase proteins, but phage Courthouse does not. Genome maps show that the nucleotide identity of the start and end of the proteins is high. All five insertions appear to encode HNH endonucleases and are likely inteins. The five insertions are not identical; Omega and LittleE have one type of intein (red boxes), while Thibault, Optimus and BAKA have a different intein (light blue boxes). (PDF) [file pone.0069273.s004.pdf]

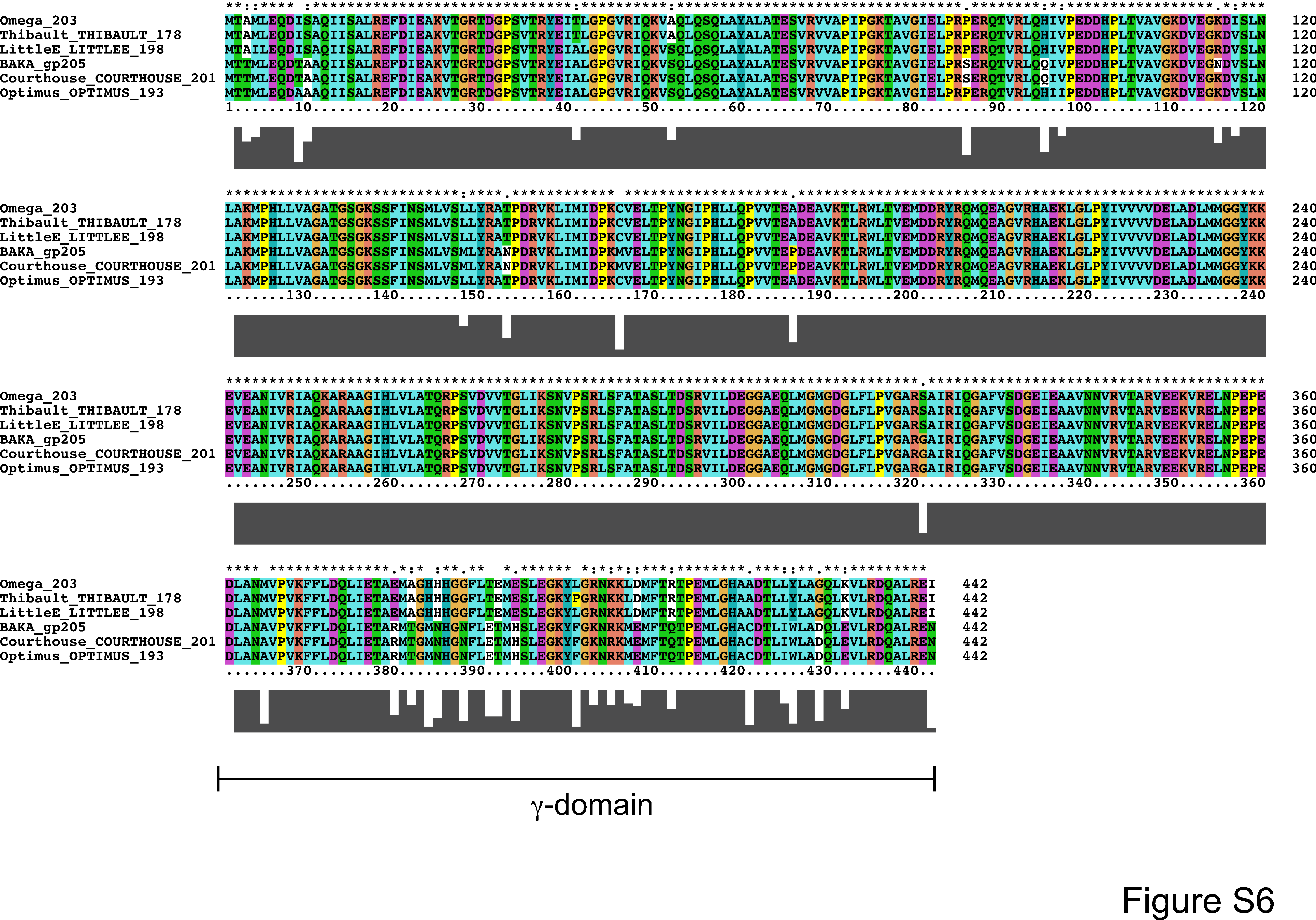

Supplement: Figure S6 — The six proteins are highly conserved in sequence, with exception of the portion that likely corresponds to the DNA-binding gamma domain. These residues show more sequence variability, and do not match any known FtsK gamma sequences. (TIF) [file pone.0069273.s006.tif]
