# Supplementary material for: Cluster J Mycobacteriophages: Intron Splicing in Capsid and Tail Genes
Source: PLoS One. 2013 Jul 9;8(7):e69273. doi: 10.1371/journal.pone.0069273 (PMC3706429; doi:10.1371/journal.pone.0069273)
Supplement: Table S1 — Author Contributions. (PDF) [file pone.0069273.s007.pdf]

Author contributions																		
						Contributions: SEA/University of Pittsburgh categories							Contributions: Journal categories					
First Name	Middle I.	Last name	Institution	email	Role	Genome Choice Participa-tion (Yes/No)	Phage Purification Amplification DNA prep (Yes/No)	Physical analysis Ems/REDs (Yes/No)	Annotation (Yes/No)	Comparative analysis (Yes/No)	Reviewed manuscript (Yes/No)	Conceived/Designed	Performed Exp	Analysis	Provide Materials/Tools	Wrote
Welkin	H.	Pope	University of Pittsburgh	welkin@pitt.edu	faculty	NA	No	Yes	Yes	Yes	Yes	Yes	Intron determination, Mass-spec, SDS gels	Yes	No	Yes
Deborah		Jacobs-Sera	University of Pittsburgh	djs@pitt.edu	faculty	NA	Yes	NA	Yes	Yes	Yes	No	Immunity Assay	Yes	No	No
Aaron	A.	Best	Hope College	best@hope.edu	faculty	Yes	Yes	Yes	Yes	Yes	Yes	Yes	Comparative analysis		Yes	No
Gregory	W.	Broussard	University of Pittsburgh	gwb9@pitt.edu	post-doc	NA	No	NA	No	No	Yes	No	Ku/Lsr2 protein work	Yes	No	No
Pamela	L.	Connerly	Indiana University Southeast	pconnerl@ius.edu	collaborator	NA	Yes	Yes	Yes	No	Yes	No	None	Yes	Yes	No
Rebekah	M.	Dedrick	University of Pittsburgh	dedrick@pitt.edu	post-doc	NA	No	NA	No	No	Yes	Yes	Integration	Yes	No	No
Timothy	A.	Kremer	Indiana University Southeast	takremer@umail.iu.edu	undergraduate student	NA	No	No	Yes	No	Yes	No	None	Yes	No	No
Susan		Offner	Lexington High School, Lexington, MA 02421	soffner@ix.netcom.com	HS teacher	NA	No	No	Yes	No	Yes	No	None	Yes	No	No
Amenawon	H	Ogiefo	University of Pittsburgh	aogiefo@bu.edu	undergradaute student	NA	Yes	Yes	No	Yes	Yes	No	Intron determination	No	No	No
Marie	C.	Pizzorno	Bucknell	pizzorno@bucknell.edu	faculty	Yes	Yes	Yes	Yes	Yes	Yes	No	None	Yes	Yes	No
Kate		Rockenbach	University of Pittsburgh	kdr25@pitt.edu	undergradaute student	NA	No	NA	No	No	Yes	Yes	Integration	Yes	No	No
Daniel	A.	Russell	University of Pittsburgh	dar78@pitt.edu	faculty	NA	No	NA	No	No	Yes	No	Sequencing	Yes	No	No
Emily	L.	Stowe	Bucknell	estoweva@bucknell.edu	faculty	Yes	Yes	Yes	Yes	Yes	Yes	No	None	Yes	Yes	No
Joseph		Stukey	Hope College	stukey@hope.edu	faculty	No	No	No	Yes	Yes	Yes	Yes	Comparative analysis		Yes	No
Sarah	A.	Thibault	Bucknell	sat019@bucknell.edu	student	Yes	Yes	Yes	Yes	Yes	Yes	No	None	Yes	No	No
James	A.	Conway	University of Pittsburgh	jxc100@pitt.edu	faculty	NA	No	NA	No	No	Yes	Yes	Cryo-EM	Yes	No	No
Roger	W.	Hendrix	University of Pittsburgh	rhx@pitt.edu	faculty	NA	No	NA	No	No	Yes	Yes	None	Yes	No	Yes
Graham	F.	Hatfull	University of Pittsburgh	gfh@pitt.edu	faculty	NA	No	NA	Yes	Yes	Yes	Yes	None	Yes	Yes	Yes

Table S1
