# Supplementary material for: Cluster J Mycobacteriophages: Intron Splicing in Capsid and Tail Genes
Source: PLoS One. 2013 Jul 9;8(7):e69273. doi: 10.1371/journal.pone.0069273 (PMC3706429; doi:10.1371/journal.pone.0069273)
Supplement: Figure S5 — Top row of the alignment is a secondary structure prediction, with upper case letters indicating more reliable predictions. The second line is the omega major capsid protein sequence and the third is the consensus hidden markov model prediction. The bottom rows are the subject rows, with the top one being the consensus hmm prediction, then the HK97 sequence, followed by two different secondary structure predictions. The central portion indicates areas of identity and similarity between the two hmm predictions. The black arrows indicate the site at which the protease cuts the major capsid proteins to remove the N-terminal delta domain from the mature capsid. The red arrow indicates the lysine residue responsible for the crosslinking of the mature major capsid proteins in HK97, which are absent from the Omega sequence. (PDF) [file pone.0069273.s005.pdf]

```
Q ss_pred                HHHHHHHHHHHHHHHHHHHHHHHHhhhcc---ccccCCCHHHHHHHhhhcchhHHHHHHHHHHHHHHHHHHHHHHHHHhhhhhh
Q Tue_Sep_11_13:      9  RALRAAAVEAVATLKAERQAIADGAKA---EERAALSADETAEFRAKSASIKAELDKVEDLDEQIRELESEIERSGKLEA   85 (478)
Q Consensus           9  ---l--L-------------l-----------r--lt-ee-------------l--e---l---i---e----------   85 (478)
                         +++|++|+++|++++++++++++++++   ++|  +||+|++++|++         +++|++.|+++|+++|++.++......
T Consensus           1  m--i--l-----------------------e----lt-e-----d-------l--e---l---i---e----------   72 (385)
T 3p8q_A              1  MSELALIQKAIEESQQKMTQLFDAQKAEIESTG-QVSKQLQSDLMK-------VQEELTKSGTRLFDLEQKLASGAENPG   72 (385)
T ss_dssp                --------------------------------------------------------------------------------
T ss_pred                CchHHHHHHHHHHHHHHHHHHHHHHHHHHHHhcC-CCCHHHHHHHHH-------HHHHHHHHHHHHHHHHHHhCCCCCCc

Q ss_pred                cccccCCCccccCcccccccchhhHHHHHHHHHHhhcCChHHHHHHhhhcccccccccHHHHHHHHHH--HhhcccCCC
Q Tue_Sep_11_13:     86  ETKTVRKATVEVNEALTYEKGNGQSYFRDLAMQTVGMADEPAKERLRRHMVDVESDKEIRKIAKVGEEY--RDLDRNGGT  163 (478)
Q Consensus          86  --------------------------------g------------------l----r-----e---ra-------  163 (478)
                         +...........                .++.  |..+  |++..                          .+.   ++++.++++
T Consensus          73  ------------------------------rg-------------------------------------------  111 (385)
T 3p8q_A             73  EKKSFSERAAEE-------------LIKS-WDGK-QGTFG-------------------------AKTFNKSLGSDADS  111 (385)
T ss_dssp                --------------------------------------------------------------------------------
T ss_pred                ccccHHHHhhhH-------------HHHH-HHhc-cCCcc-------------------------cccchhhhccccCC

Q ss_pred                CccccChhhhhHHHHHHHHhcchHHHhhcceEEecCCcccEEEEEEccccceeEEeccCCcCCCccccccCCCceeEEEech
Q Tue_Sep_11_13:    164  GGYAVPPLWMMNRFIELARAGRTYANLCPTEPLPGGTSSINIPKILTGTSTAIQAADNAALTAPSAHEVDLTDGFVQANV  243 (478)
Q Consensus         164  gg-lvP-------i---------l--l----------g----p--------a--v-Eg-----s---------f--v----  243 (478)
                         +|.+||+++. +.|++.+++.++|+++|+++|++++.  ++||+.++.++.|.||+||+.+|++        +++|+++++++
T Consensus         112  -g-lvp------ii---------l--l--------------------------a--v-Eg------------f--v----  183 (385)
T 3p8q_A            112  AGSLIQPMQI-PGIIMPGLRRLTIRDLLAQGRTSSNA--LEYVREEVFTNNADVVAEKALKPES-----DITFSKQTANV  183 (385)
T ss_dssp                -------CBC-CCCCCCCCSSCCHHHHSEEEECCSSE--EECCCCEECCCCSSSCCEECCCCEE-----ECEEEEEEEEB
T ss_pred                CCccccChhhh-HHHHHhhhchhhHHHHhcccccCCCC--EEEEEEcCCCccceEecCCCCcCcC-----ccceEEEEeee

Q ss_pred                hheeEehHhhHHHHhhhhhhhHHHHHHHHHHHHHHHHHHHHHheeCCCCCCCCcccccccccccccccCCccchhhhhhhHHH
Q Tue_Sep_11_13:    244  KTIAGQQGIAIQLLDQAAVSVDEFVFRDLAADYANKLNVQVISGTGSNNQVVGVRATAGITQVTATSAGSALEKHQIIYQ  323 (478)
Q Consensus         244  -k------iS-ell-ds---l---v---la-a-a---d---l-G-G----p-Gi---------v------------------  323 (478)
                         ++++++++||+|+|+|+ ++++++|.++|+++++++++|.+||+|+|++..|+||++.++......+.++...          ++
T Consensus         184  -k------iS-ell-d----l---i---la-------d--il-G-G------Gi-------------------------d  256 (385)
T 3p8q_A            184  KTIAHWVQASRQVMDDA-PMLQSYINNRLMYGLALKEEGQLLNGDGTGDNLEGLNKVATAYDTSLNATGDTR------AD  256 (385)
T ss_dssp                CHHHHTTSTHHHHHHHHT-THHHHHHHHHHHHHHTTCCCSTTCCCCHHHHSEECCTTSCCSSCCHHHHHHHHHH------HT
T ss_pred                EEEEEeccCCHHHHhCh-HHHHHHHHHHHHHHHHHHHHhhhhhcCCCCCCCCcccccCccccccccCCCch------HH

Q ss_pred                HHHHHHhhhhhhhhccccCcEEEECHHHHHHHHHHhhccCCCEEEeeCcccccccccccccccccCCcceEcceeEEEeCCCCCc
Q Tue_Sep_11_13:    324  KIADAIQRVHTSRFLEPEVIVMHPRRWASFHAIFAGDDRPLIVPSGPGFNNLGVLTEVASQRVVGQMHGLPVVTDPTLPT  403 (478)
Q Consensus         324  -l--a---l-----------im-------l--lkd--G--l--p--------g---g----g----l-G-PV------p-  403 (478)
                         +|+++++++++.+..|..++  |+|||++|..|+++|||++|+|+|+|+ +..                .+.+++|+|+|||+++++||.
T Consensus         257  -l------l-----------vmn------l--lkd--G--l------------------------------l-G-PV--------  321 (385)
T 3p8q_A            257  IIAHAIYQVTESEFSASG-IVLNPRDWHNIALLKDNEGRYIFG-GPQ-------------AFTSNIMWGLPVVPTKAQAA  321 (385)
T ss_dssp                TTTTCCCCEEECCHHHHH-HHHSCCCSSSCCSSCTTTTTSSSE-ETT-------------EEECCCSSSCSSCCEEECTT
T ss_pred                HHHHHHHHHHhcCCCCcE-EEECHHHHHHHHHHhcccCCCEeec-ccc-------------cCCCceeCCeeEEecCCCCC

Q ss_pred                cccCCCCCceEEEEEccc-CEEEeccC-ceEEeec--cccCCCeEEEEEEeeecccccCcccEEEEecce
Q Tue_Sep_11_13:    404  TLGTGTDQDVIHVLRASD-LALFESSV-RMRALQE--TRAENLSVLLQVYGYLAFTAARFPQSVVEIGGTA  470 (478)
Q Consensus         404  --g---d------gd----i-----------d-------------rv--------v----a-v------  470 (478)
                         +    .     ++||||+. +.++++++ .+.+..+  .++.+++.||++.|+++ ++.+|+|||++++...
T Consensus         322  g-----------Gdf-----i--r----v-----------------v-------r-d--v----A---l----  383 (385)
T 3p8q_A            322  G----T----FTVGGFDMASQVWDRMDATVEVSREDRDNFVKNMLTILCEERLAL-AHYRPTAIIKGTFSS  383 (385)
T ss_dssp                T----S----CEEEEEEEEEEEEESCSSSTTTTTCCEEEEEEEEEEECSCGGGEE-ECCCCC---------
T ss_pred                C----e----EEEEEhhhCEEEEEeCCcEEEEeccCCCcccccCeEEEEEEEEEee-eecCCCcEEEEEecC
```

Figure S5
